# Reduced replication fork speed promotes pancreatic endocrine differentiation and controls graft size

**DOI:** 10.1172/jci.insight.141553

**Published:** 2021-03-08

**Authors:** Lina Sui, Yurong Xin, Qian Du, Daniela Georgieva, Giacomo Diedenhofen, Leena Haataja, Qi Su, Michael V. Zuccaro, Jinrang Kim, Jiayu Fu, Yuan Xing, Yi He, Danielle Baum, Robin S. Goland, Yong Wang, Jose Oberholzer, Fabrizio Barbetti, Peter Arvan, Sandra Kleiner, Dieter Egli

**Affiliations:** 1 Naomi Berrie Diabetes Center, Columbia University, New York, New York, USA.; 2 Department of Pediatrics, Department of Obstetrics and Gynecology, Columbia Stem Cell Initiative, Columbia Irving Medical Center, Columbia University, New York, New York, USA.; 3 Regeneron Pharmaceuticals, Inc., Tarrytown, New York, USA.; 4 Bambino Gesù Children’s Hospital, Rome, Italy.; 5 Division of Metabolism, Endocrinology & Diabetes, Department of Internal Medicine, University of Michigan, Ann Arbor, Michigan, USA.; 6 PhD program in the Department of Physiology and Cellular Biophysics, Columbia Irving Medical Center, Columbia University, New York, New York, USA.; 7 Department of Surgery, University of Virginia, Charlottesville, Virginia, USA.

**Keywords:** Cell Biology, Stem cells, Beta cells, Cell cycle, Stem cell transplantation

## Abstract

Limitations in cell proliferation are important for normal function of differentiated tissues and essential for the safety of cell replacement products made from pluripotent stem cells, which have unlimited proliferative potential. To evaluate whether these limitations can be established pharmacologically, we exposed pancreatic progenitors differentiating from human pluripotent stem cells to small molecules that interfere with cell cycle progression either by inducing G_1_ arrest or by impairing S phase entry or S phase completion and determined growth potential, differentiation, and function of insulin-producing endocrine cells. We found that the combination of G_1_ arrest with a compromised ability to complete DNA replication promoted the differentiation of pancreatic progenitor cells toward insulin-producing cells and could substitute for endocrine differentiation factors. Reduced replication fork speed during differentiation improved the stability of insulin expression, and the resulting cells protected mice from diabetes without the formation of cystic growths. The proliferative potential of grafts was proportional to the reduction of replication fork speed during pancreatic differentiation. Therefore, a compromised ability to enter and complete S phase is a functionally important property of pancreatic endocrine differentiation, can be achieved by reducing replication fork speed, and is an important determinant of cell-intrinsic limitations of growth.

## Introduction

The unlimited proliferation potential of human pluripotent stem cells is both an opportunity as well as a challenge: it provides a renewable source of cells for cell replacement for degenerative disorders such as diabetes, but it is also a risk for the formation of growths in cell transplants. Limitations in proliferation potential are established in parallel to limitations in differentiation potential; cells of the adult pancreas have largely stable identities and a very low proliferative and regenerative potential. Limitations in cell proliferation of the pancreas are cell intrinsic and established during embryonic expansion of pancreatic progenitors (1). Proliferation of β cells in the developing human pancreas occurs primarily during embryogenesis, and declines after birth, and proliferation in adult β cells is essentially absent (2, 3). During terminal differentiation, many cell types, including neurons and muscle cells, exit the cell cycle as they adopt full functionality (4, 5). When forced into the cell cycle, adult β cells and neurons frequently undergo apoptosis (6, 7), suggesting a compromised ability to progress and complete S phase. Whether these limitations in S phase progression play a functional role in establishing cell-intrinsic limitations in cell proliferation and are important to establish the terminally differentiated state is not known.

Several studies show that cell cycle progression can be disruptive to β cell function. When β cells are immortalized to generate proliferating β cell lines, the differentiated state and function are compromised. For instance, the stable transformed mouse insulinoma cell line MIN6 and the rat insulinoma cell line INS1E develop glucose-independent insulin secretion and express other islet hormones with increasing passage (8–10). Overexpression of the pro-proliferation molecules cyclin-dependent kinases in primary rat β cells increases proliferation, leads to dedifferentiation of primary β cells, and reduces glucose-stimulated insulin secretion (11). Furthermore, overexpression of the oncogene c-Myc in adult mouse β cells increases proliferation and β cells acquire an immature phenotype (12). Conversely, removal of immortalizing transgenes in EndoC-βH1 cell line, a proliferative immortalized β cell line generated from human fetal pancreas, decreases cell proliferation and enhances β cell–specific features, such as increased insulin gene expression and content (13). Furthermore, several studies show that cell cycle progression is disruptive to the differentiated state in general: cell cycle progression plays a critical role in mediating the transition of a differentiated cell to a pluripotent stem cell (14–20). Oncogenic principles disrupting the limitations in cell proliferation of a differentiated cell can facilitate reprogramming, including mutations in *P53* (21, 22) or *Rb* (23), as well as *C-myc* (24) or SV40 T-Ag overexpression (25). These manipulations directly affect DNA replication and/or impair the exit from the cell cycle in response to genome instability.

We reasoned that the reverse might also apply: that limitations in cell cycle progression can be established through interference with the progression of DNA replication and that these manipulations promote differentiation to insulin-producing cells from pluripotent stem cells and help establish cell-intrinsic limitations in growth potential. To test this, we treated pancreatic progenitors with compounds interfering with DNA replication and/or cell cycle progression through different mechanisms and determined β cell differentiation efficiency, stability of the differentiated state, and β cell function in vitro and in vivo. Compounds that interfered with G_1_ to S phase transition, as well as with S phase completion, were most effective in increasing differentiation efficiency to insulin-producing cells, resulted in greater stability of the differentiated state, and increased the robustness of the differentiation protocol with high endocrine cell yield. These compounds included the DNA polymerase inhibitor aphidicolin, the antineoplastic agent cisplatin, and the topoisomerase inhibitor etoposide. Inducing G_1_ arrest alone, such as through CDK4 inhibition without compromising transition through S phase, was less effective. We found that endocrine induction signaling pathways can be substituted through inhibition of DNA replication, suggesting that a primary mechanism in the activity of these pathways is to affect DNA replication and cell cycle progression. Upon transplantation, aphidicolin-treated insulin-producing cells demonstrated higher human C-peptide secretion, demonstrated greater responsiveness to glucose level changes, and protected mice from diabetes without the formation of teratomas or cystic structures. These results demonstrate that limitations in DNA replication link proliferation potential and β cell identity, which can be exploited to improve graft outcomes in the context of cell replacement for diabetes.

## Results

### Aphidicolin reduces DNA replication fork speed in a dose-dependent manner in pancreatic progenitors.

**Figure 1:**
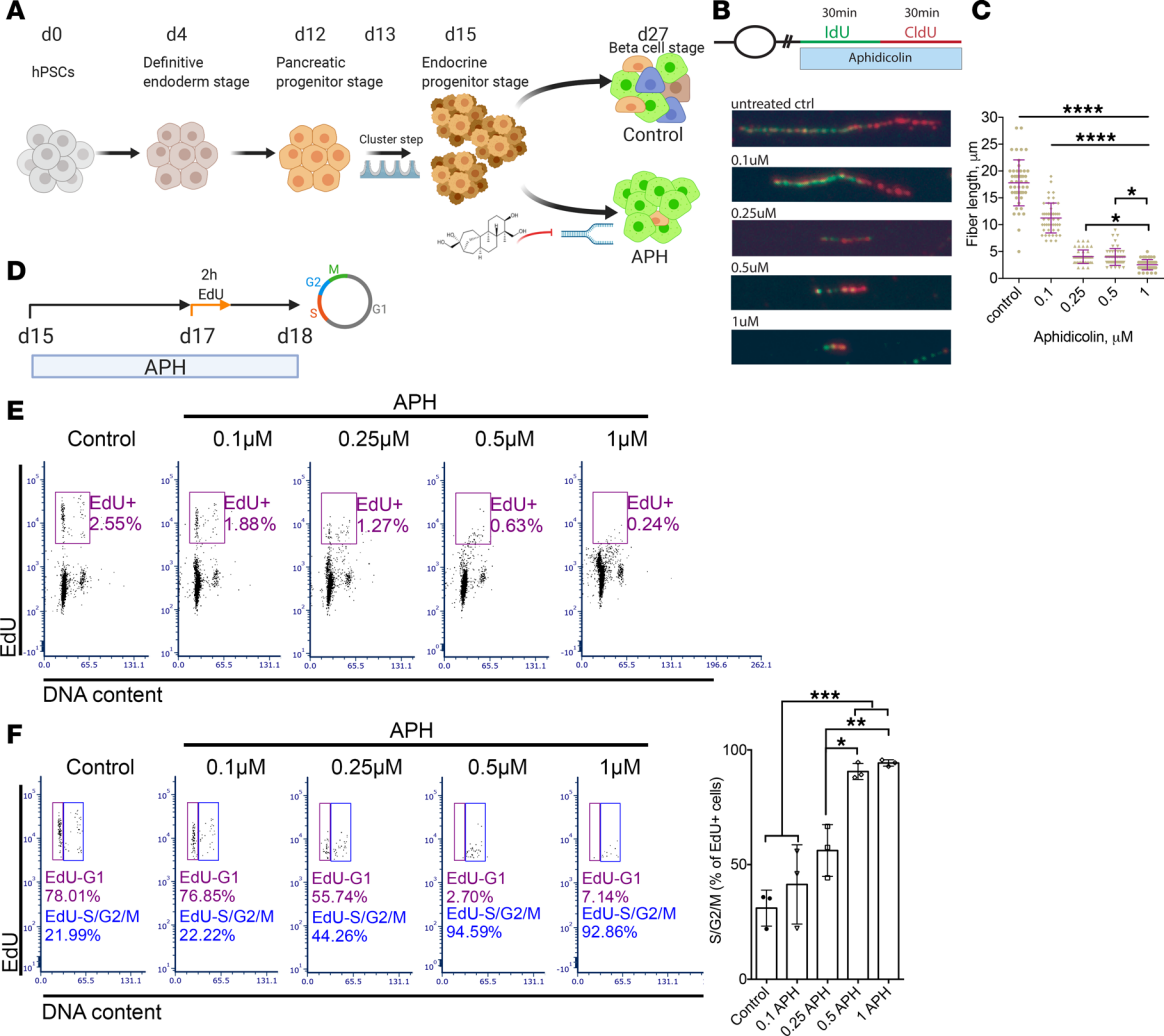
APH affects DNA replication progression in a dose-dependent manner in pancreatic progenitors. (**A**) A schematic diagram represents the differentiation of human pluripotent stem cells toward insulin-producing cells with and without APH treatment. Created with BioRender.com. (**B**) DNA replication progression analysis by labeling cells with IdU and CIdU and (**C**) quantification of labeled fiber length. One-way ANOVA with *P < 0.05; ****P < 0.0001. All conditions under **** are significantly different from control or 0.1 μM. (**D**) A schematic diagram indicates the time of APH treatment, EdU incubation, and cell cycle analysis. Created with BioRender.com. (**E**) A representative flow plot of 3 independent experiments showed the cell cycle profile of cells treated with different concentrations of APH on day 18 after 2 hours of EdU labeling on day 17. (**F**) The percentage of EdU-positive cells in S/G_2_/M phase and that failed to progress to G_1_ phase was determined on day 18 (*n* = 3). One-way ANOVA test with *P < 0.05; **P < 0.01; ***P < 0.001.

Aphidicolin (APH) is a DNA polymerase inhibitor interfering with DNA replication fork progression with dose-dependent effects on S phase entry and completion (26, 27). At high concentrations, APH inhibits S phase entry, while at low concentrations, APH inhibits S phase completion and enhances fragility of common fragile sites while S phase entry is not impaired (28). To understand the effect of S phase entry and completion on pancreatic differentiation from pluripotent stem cells, we exposed cells to different concentrations of APH from the pancreatic endocrine progenitor stage (day 15) to the β cell stage (day 27) (Figure 1A). Cells were exposed to APH from day 15 to day 27 at increasing concentrations from 0.1 μM to 1 μM. We evaluated replication progression by sequentially labeling the cells with thymidine analogs iodo-deoxyuridine (IdU) and chloro-deoxyuridine (CIdU) for the first hour of APH on day 15 (Figure 1B). Replication fork speed decreased from 1.6 kb per minute in untreated cells to 0.5 kb per minute in cells treated with 0.25 μM or 0.5 μM and was further reduced at 1 μM APH (Figure 1C), consistent with earlier studies in other cell types (27). The decrease in replication fork progression correlated with cell cycle progression examined on day 18 with 2-hour ethinyl-deoxyuridine (EdU) labeling on day 17 (Figure 1, D and E). Most cells arrested in G_1_ phase, and less than 1% of cells were in S phase when treated with 0.5 μM and 1 μM APH (Figure 1E). When cells were pulsed with EdU for 2 hours and released to complete the cell cycle (Figure 1D), control cells progressed to G_1_ phase, as indicated by EdU-positive cells in G_1_ (Figure 1E). In contrast, with increasing concentrations of APH, pancreatic progenitor cells showed a delayed replication progression, indicated by the high percentage of EdU-positive cells that were in G_2_/M phase in the presence of a concentration of 0.25 μM (56.1% ± 11.3%), 0.5 μM (90.6% ± 3.5%), and 1 μM APH (94.3% ± 1.4%) compared with control (31% ± 7.8%) and 0.1 μM APH (41.4% ± 17.3%) (*n* = 3 for each condition). Lower concentration of 0.1 μM APH allowed S phase entry and the progression through S phase (Figure 1F).

### APH promotes pancreatic endocrine cell differentiation from stem cells and reduces the variability of differentiation in vitro.

**Figure 2:**
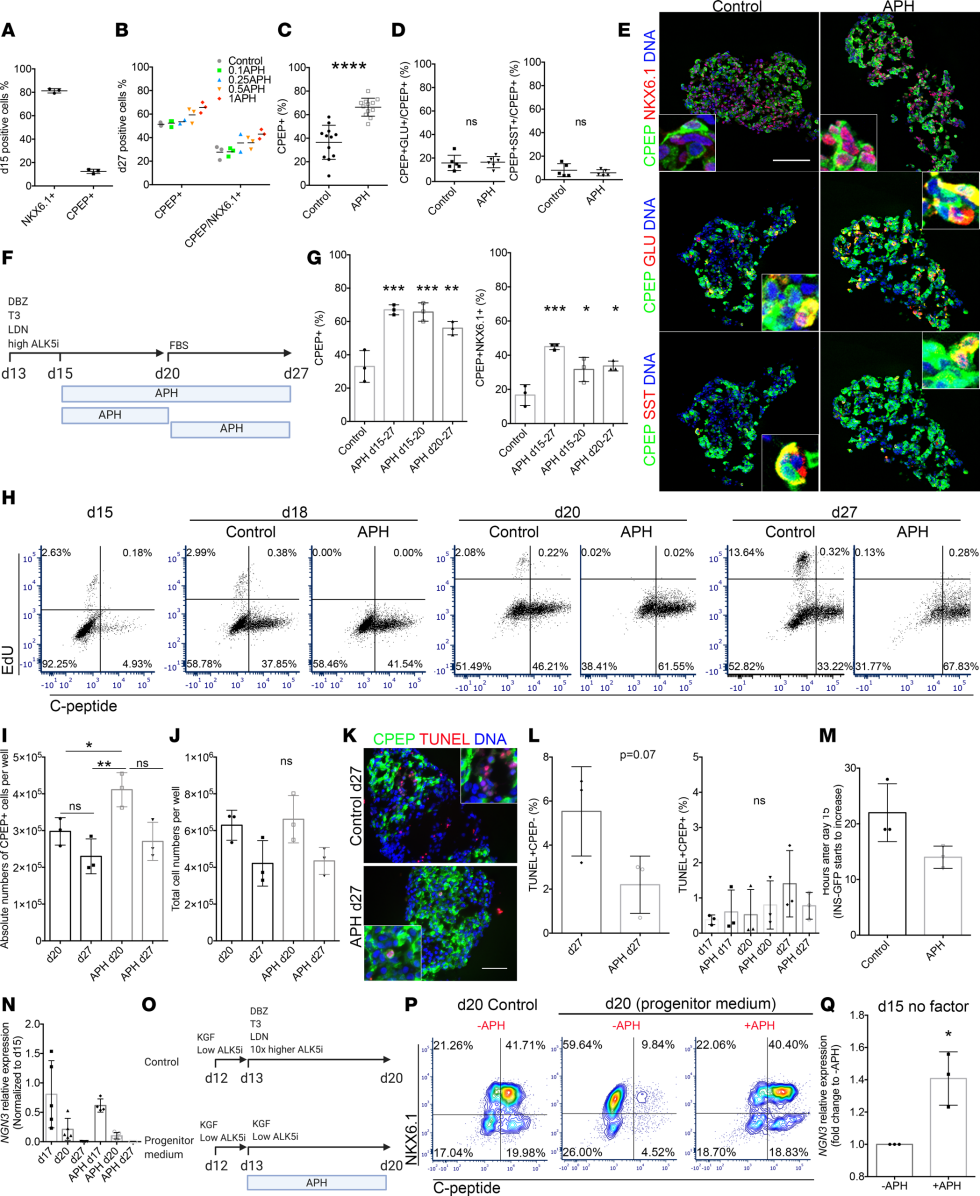
Inhibiting replication fork progression promotes endocrine differentiation independent of apoptosis. (**A**) Quantification of NKX6.1 or C-peptide–positive cells on day 15 (*n* = 3) and on day 27 (**B**) with different concentrations of APH. (**C**) Quantification of C-peptide–positive cells in control and 1 μM APH on day 27 (*n* = 12). Two-tailed paired *t* test with ****P < 0.0001. (**D**) Quantification of C-peptide/glucagon-positive cells (*n* = 6) or C-peptide/somatostatin-positive cells (*n* = 5). (**E**) Immunostaining of APH-treated clusters for C-peptide, NKX6.1, glucagon, and somatostatin on day 27. Scale bar: 100 μm. Insets: 6.25× higher magnification. (**F**) A schematic diagram indicates APH duration. (**G**) Flow cytometry quantification of C-peptide–positive and C-peptide/NKX6.1-positive cells in indicated conditions on day 27 (*n* = 3). One-way ANOVA test, *P < 0.05; **P < 0.01; ***P < 0.001. (**H**) Flow cytometry profile of cell cycle progression on days 15, 18, 20, and 27 without and with APH, indicated by the percentage of cells positive for C-peptide and EdU. (**I**) Quantification of total C-peptide–positive cells on days 20 and 27. One-way ANOVA test, *P < 0.05; **P < 0.01. (**J**) Total cell numbers on days 20 and 27. (**K**) Immunostaining on day 27 clusters for TUNEL and C-peptide (6.25× higher magnification in inset). Scale bar: 100 μm. Quantification of TUNEL-positive and C-peptide–negative cells on day 27. (**L**) Quantification of C-peptide– and TUNEL-positive cells on days 17, 20, and 27 (*n* = 3). (**M**) Timing of insulin-GFP expression. (**N**) *NGN3* expression determined by quantitative real-time PCR (RT-PCR). (**O**) Schematic diagram of experimental conditions. (**P**) Flow cytometry analysis of day 20 cells for NKX6.1 and C-peptide when day 13 pancreatic progenitors were cultured in progenitor medium with and without APH (day 20 control no APH: 41.5% ± 13.44%; day 20 progenitor medium no APH: 13% ± 1.4%; day 20 progenitor medium plus APH: 52% ± 9.9%, *n* = 2). (**Q**) Quantitative RT-PCR for *NGN3* expression of day 15 cells cultured in basal endocrine medium with and without APH (*n* = 3). Two-tailed unpaired *t* test, *P < 0.05. DBZ, γ-secretase inhibitor; T3, thyroid hormone; LDN, LDN193189; KGF, keratinocyte growth factor.

To determine the effect of APH on endocrine differentiation, we treated endocrine progenitor cells from day 15 to 27 with indicated concentrations of APH and quantified the differentiation efficiency. On day 15, before APH treatment, 81.3% ± 1.9% of cells expressed progenitor marker NKX6.1, and only few cells expressed C-peptide (12.5% ± 1.8%) with low intensity, indicating they were at the stage of pancreatic endocrine progenitors (Figure 2A). APH concentration of 0.1 μM had no effect on the percentage of pancreatic endocrine cells on day 27. APH concentrations starting from 0.25 μM resulted in higher differentiation efficiencies than controls, with 1 μM APH giving rise to the highest percentage of C-peptide– and NKX6.1-positive cells (Figure 2B and Supplemental Figure 1A; supplemental material available online with this article; https://doi.org/10.1172/jci.insight.141553DS1). In a total of 12 independent experiments, 66.3% ± 7.6% C-peptide–positive cells were induced in the condition with 1 μM APH, which was significantly higher than the percentage of C-peptide–positive cells generated in 12 independent controls differentiated in parallel (36.5% ± 14.5%) (*n* = 12) (Figure 2C). Similar percentage of C-peptide–positive cells coexpressed glucagon (APH: 16.5% ± 4.8%; control: 15.8% ± 6.5%, *n* = 6) or somatostatin (APH: 5.9% ± 2.6%; control: 8.1% ± 5.5%, *n* = 5) in the stem cell–derived endocrine clusters between APH and control, indicating that APH did not alter the proportion of polyhormonal cells (Figure 2, D and E, and Supplemental Figure 1B). APH treatment reduced the variability of β cell differentiation; without APH, the percentage of C-peptide–positive cells ranged from 10% to 60% (*n* = 12). With APH, all cultures contained more than 50% C-peptide–positive cells with the highest over 80% (*n* = 12) (Figure 2C). To evaluate if the effect of APH was consistent across different genetic backgrounds, we included 2 induced pluripotent stem cell (iPSC) lines with different differentiation potentials, 1018E and 1023A. 1018E was previously identified as a cell line with poor differentiation competence (29). The percentage of C-peptide–positive cells was significantly higher after APH treatment in both cell lines (Supplemental Figure 1, C and D). Remarkably, the poor differentiation potential of 1018E increased to the range of a differentiation-competent cell line, from an average of 11% to 38% (*n* = 3) (Supplemental Figure 1C). Thus, APH increases the purity of insulin-producing cells after formation of pancreatic progenitors in human embryonic stem cells and iPSCs of different genetic backgrounds.

To determine how and at which stage of differentiation APH acts to promote endocrine differentiation, we applied APH at different stages, including early stage (d15–d20) during endocrine progenitor differentiation, and after commitment of endocrine lineages (d20–d27), and evaluated the percentage of C-peptide–positive cells as well as C-peptide and NKX6.1 double-positive cells at the end of differentiation on day 27 (Figure 2F). Addition of APH at all indicated stages increased the proportion of C-peptide–positive cells as well as C-peptide and NKX6.1 dual-positive cells (Figure 2G and Supplemental Figure 1E).

We profiled cell cycle progression on day 15, day 18, day 20, and day 27 of β cell differentiation (Figure 2H). In untreated cells, C-peptide–positive cells started to form on day 15 (approximately 5%) and reached a peak on day 20 with approximately 46% C-peptide–positive cells. APH treatment improved the percentage of C-peptide–positive cells to approximately 60% (Figure 2H and Supplemental Figure 2A). Approximately 2% of all cells in control and very few cells, if any, in the APH condition underwent proliferation during 2-hour EdU incubation at each stage before day 20 (Figure 2H). Cells treated with APH had an increased number of C-peptide–positive cells on day 20 (Figure 2I), while the total cell number remained the same between the 2 groups (Figure 2J). When compared to controls with the highest differentiation efficiency, the total number of C-peptide–positive cells between APH-treated and control groups was similar on day 27. No significant apoptosis was detected in C-peptide–positive cells after APH treatment, neither at an early stage on day 17, nor at late stage on day 20 and day 27, as measured by TUNEL staining (Figure 2, K and L, and Supplemental Figure 2B). Increased apoptosis was detected in C-peptide–negative cells in the control group (Figure 2K). We also traced the expression of insulin-GFP using live-cell imaging starting from day 15 when APH was added. APH-treated progenitor clusters started to express insulin 8 hours earlier than clusters without APH: GFP started to increase at 14.00 ± 1.16 hours in APH and 22.00 ± 3.00 hours in control condition (Figure 2M and Supplemental Videos 1–6), and insulin-GFP glowed brighter in APH-treated clusters compared with control. Thus, the increased percentage of C-peptide–positive cells from day 15 to day 20 is due to increased differentiation, not due to inhibiting the expansion of other cell types and not due to cell death of C-peptide–negative cells.

To test if the increased endocrine differentiation efficiency is due to the maintenance of neurogenin 3 (*NGN3*) expression in APH-treated cells, we checked the transcription levels of *NGN3* in control and in APH-treated cells on days 15, 17, 20, and d27. *NGN3* is an essential transcription factor expressed in endocrine progenitors. We found that the average expression of *NGN3* decreased from day 15 to 17 in both conditions, but control cells showed a greater variability in *NGN3* expression levels among each batch of differentiated cells (*n* = 5) compared with APH groups (*n* = 5) (Figure 2N). This variability might contribute to the variability in the final percentage of C-peptide–positive cells ranging from 10% to 60% in controls (Figure 2C) and consistently above 50% in APH-treated samples.

To explore the effect of reduced replication fork speed on the differentiation of duodenal homeobox 1 (PDX1) and NKX6.1 double-positive pancreatic progenitor to insulin-producing cells, we cultured cells with APH starting from the pancreatic progenitor stage on day 13 (2 days before pancreatic endocrine progenitor stage) to day 20 in pancreatic progenitor medium (Figure 2O). NKX6.1-positive cells failed to progress to C-peptide–positive cells in progenitor medium (Figure 2P). In stark contrast, when APH was added, it resulted in the efficient induction of C-peptide–positive cells, comparable to cell culture with endocrine induction medium containing thyroid hormone T3, TGF-β receptor 1 inhibitor (ALK5i), and bone morphogenetic protein 4 inhibitor, and Notch signaling (control) (Figure 2P). To specifically test if APH can replace these endocrine induction factors, we removed them from the differentiation medium, and instead added APH, and tested for the induction of NGN3. *NGN3* expression was significantly increased on day 15 after 2 days of APH addition without any induction factors, compared with cells cultured in the absence of APH (Figure 2Q). These results show that the reduction of DNA replication fork speed is sufficient to promote pancreatic endocrine differentiation from progenitors and suggest that endocrine induction signaling pathways may be upstream of processes acting at the level of DNA replication.

Next, we determined how APH increased the percentage of C-peptide–positive cells after commitment to the endocrine fate. A high percentage of C-peptide–positive cells was maintained from day 20 to day 27 in the APH condition but declined in controls (Figure 2H). The decline in control cells was primarily driven by cell proliferation of insulin-negative cells: from day 20 to day 27, approximately 13% of C-peptide–negative cells showed proliferation in controls and essentially none in APH-treated samples. This suggests that at late stage of differentiation, APH maintains C-peptide–positive cells mainly through the inhibition of proliferation of nonendocrine cell types. The total number of cells in control was not significantly different from that in APH-treated condition (Figure 2J). This may be due to the increased apoptosis of C-peptide–negative cells observed in controls (Figure 2K). In summary, APH improves differentiation efficiency both by promoting endocrine differentiation and by preventing expansion of nonendocrine cells when endocrine differentiation factors are no longer applied.

### Inhibition of S phase entry and compromised S phase completion promote endocrine differentiation.

**Figure 3:**
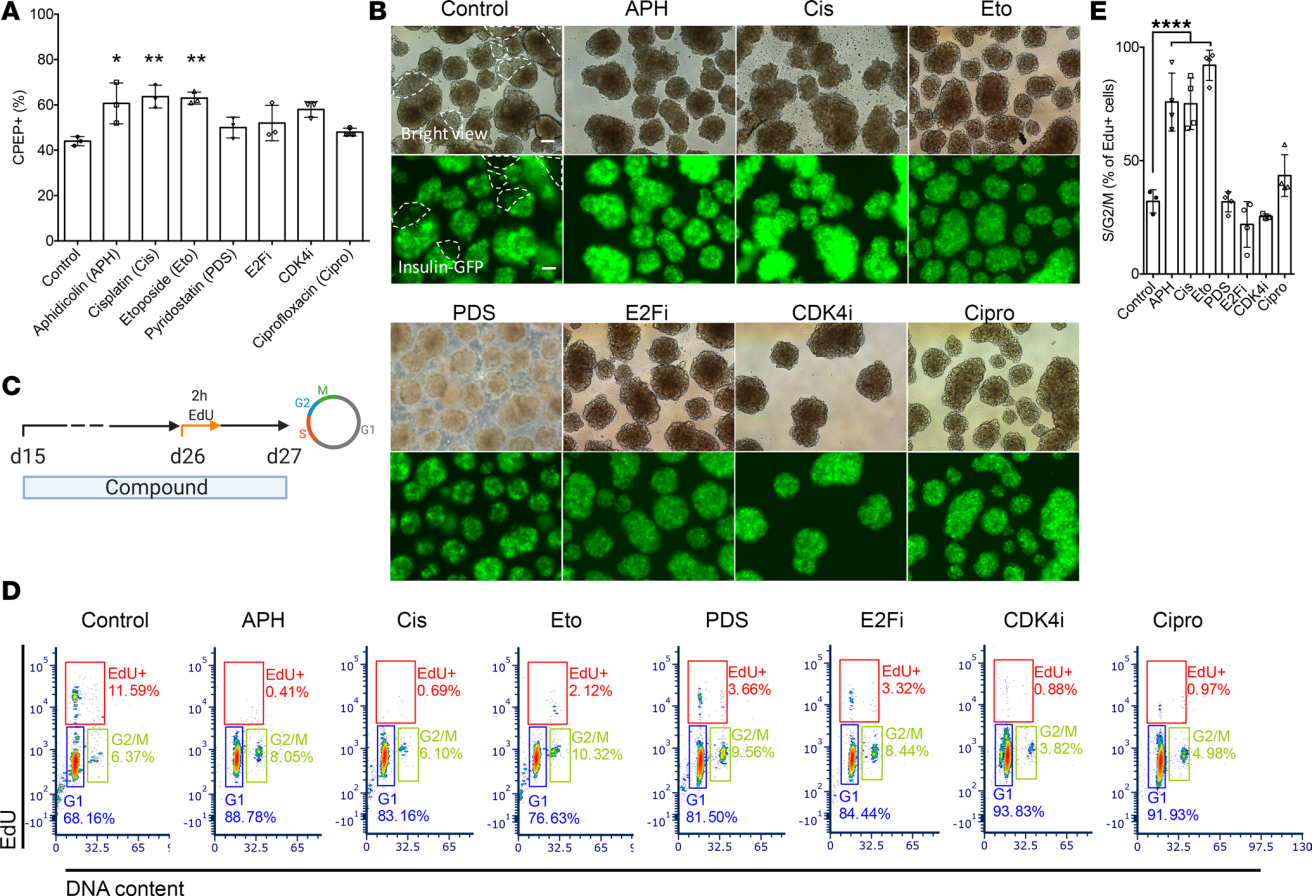
Interference with DNA replication completion promotes differentiation of insulin-producing cells. (**A**) Flow cytometry quantification of C-peptide–positive cells on day 27 with indicated compounds (*n* = 3). One-way ANOVA test *P < 0.05; **P < 0.01. (**B**) Representative bright view and fluorescence picture of stem cell–derived islet clusters after treatment with indicated compounds. GFP-negative parts are circled by dashed white line. Scale bar: 100 μm. Pictures were taken with an OLYMPUS IX73 fluorescence microscope with equal exposure time (148.1 ms). (**C** and **D**) EdU pulse and chase experiment to determine S phase entry and progression. Cells treated with indicated compounds were labeled with EdU for 2 hours on day 26 and analyzed 1 day later for cell cycle distribution. (**D**) A representative flow plot showed percentage of cells in each cell cycle. (**E**) Quantification of EdU-positive cells in S/G_2_/M phase (*n* = 3–4). One-way ANOVA test ****P < 0.0001.

To determine whether the increased differentiation efficiency is specific to polymerase inhibition, or due to its effect on S phase entry and/or progression, we tested a panel of compounds interfering with either S phase entry or completion or both. The cyclin-dependent kinase 4 inhibitor (CDK4i) arrests cells in early G_1_ phase (30). The MCM replicative helicase inhibitor (ciprofloxacin) and the E2F inhibitor (E2Fi), a transcription factor required for regulating expression of S phase genes (31), prevent entry into S phase. Cisplatin (Cis) induces DNA damage by cross-linking DNA, interferes with S phase progression, and arrests cells at G_0_/G_1_ phase (32); etoposide (Eto) is a topoisomerase II inhibitor inhibiting the unwinding of the DNA helix during replication and transcription and arrests cells mainly in the S and G_2_ phases (33). Other tested compounds include pyridostatin (PDS), a compound promoting the formation of G_4_ structures to induce replication fork stalling (34), thereby affecting S phase progression. The percentage of C-peptide–positive cells was increased in all conditions treated with the indicated compounds in comparison with untreated controls (*n* = 3) (Figure 3A and Supplemental Figure 3, A and B). A high percentage of insulin-expressing cells indicated by the expression of GFP and evenly distributed in the islet-like clusters were observed with all compounds tested, whereas some parts of the clusters in control remained GFP negative (Figure 3B). Notably, cells treated with APH and Cis expressed higher levels of GFP compared with cells treated with other compounds and control.

We analyzed the cell cycle progression by labeling cells with EdU for 2 hours on day 26 and collected cells for flow cytometry analysis the next day (Figure 3C). We found that the ability to increase the percentage of insulin-positive cells correlated with an increase in the percentage of G_1_/G_0_ cells, a reduction of EdU-positive cells, as well as an increase in the percentage of G_2_/M cells relative to cells that completed the cell cycle in the presence of the compound and progressed to G_1_ (*n* = 3–4) (Figure 3, D and E, and Supplemental Figure 3C). APH, cisplatin, and etoposide resulted in all 3 changes to cell cycle progression (Figure 3, D and E, and Supplemental Figure 3C). Compounds or concentrations that did not fulfill all 3 changes were less effective. CDK4i and the replication licensing inhibitor ciprofloxacin arrested cells in G_1_ with comparable efficiency to APH but showed only an insignificant increase in endocrine differentiation (Figure 3, A and D). CDK4i had no effect on the progression of replicating cells through G_2_ to G_1_ phase (Figure 3E). E2Fi and PDS reduced the number of cells in S phase but not to the same extent as other compounds (Figure 3D). Consistent with compromised S phase completion, APH concentrations 0.25–1 μM resulted in a significant increase in 53BP1 bodies relative to controls (Supplemental Figure 4). 53BP1 bodies mark incompletely replicated DNA inherited during cell division (35, 36). Such incomplete replication promotes the decision to enter quiescence (37). Control cells also showed low levels of 53BP1 bodies, which were not increased by treatment with CDK4i. Cisplatin increased both 53BP1 bodies as well as DNA damage marked by γH2AX relative to both APH-treated cells as well as controls (Supplemental Figure 4). Thus, induction of G_1_ arrest and compromised completion of S phase progression is an intrinsic property of pancreatic differentiation and can be used to promote differentiation to stem cell–derived insulin-producing cells, measured by the increased percentage of C-peptide–positive cells.

### Transcriptome analysis shows an increase in endocrine cells and a decrease in nonendocrine cells.

**Figure 4:**
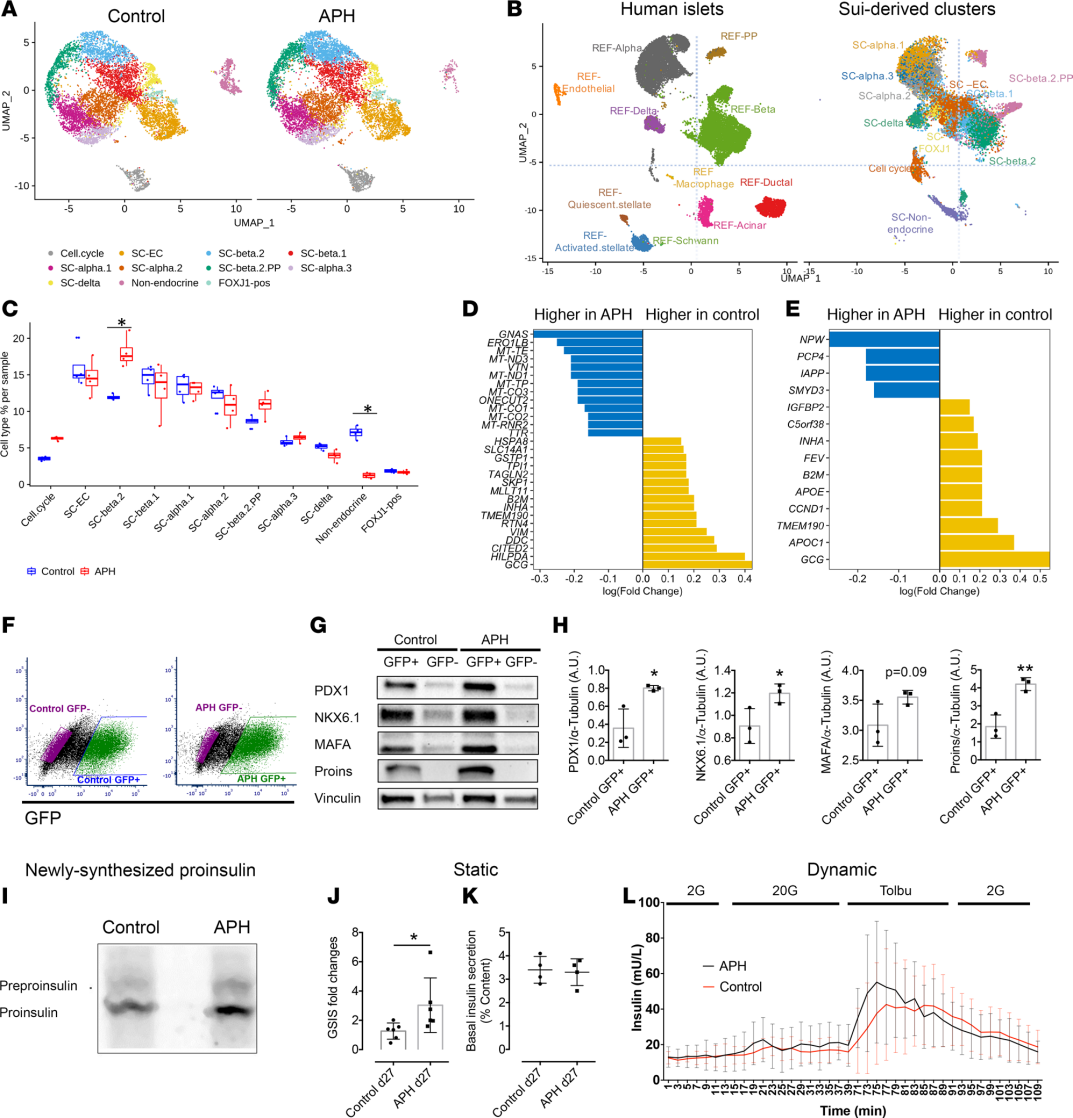
Single-cell transcriptome analysis shows an increase of endocrine cells and decrease of nonendocrine cells in the APH condition. (**A**) Identified cell populations in stem cell–derived islet cells with and without APH during differentiation (samples were collected from 4 independent wells of 1 experiment in each condition). (**B**) Identified cell populations in stem cell–derived islet cells compared with primary human islet cells. (**C**) Quantification of indicated cell populations between control and APH groups. *Wilcoxon’s test with *P* < 0.05. (**D**) The upregulated and downregulated genes after cells were treated with APH compared with control in SC-β 1 cells and (**E**) SC-β 2 cells. (**F**) Insulin-GFP–positive and –negative cells were sorted from control and APH groups for downstream analysis. (**G**) Protein expressions of sorted cells by a representative Western blot analysis for PDX1, NKX6.1, MAFA, Proins, and Vinculin (Supplemental Figure 9, A and B). (**H**) Quantification of Western blot band intensity and normalized to α-tubulin (*n* = 3). Two-tailed paired *t* test *P < 0.05; **P < 0.01. (**I**) Proinsulin biosynthesis of an iPSC line–derived β cell clusters with and without APH treatment (Supplemental Figure 9C). (**J**) Static glucose stimulated insulin secretion (GSIS) (a stimulation index is determined by the fold changes of insulin secretions of nonsorted cells incubated in 2 mM and 20 mM glucose). Mann-Whitney *U* test *P < 0.05. (**K**) Basal insulin secretion levels of nonsorted cells normalized to insulin content. (**L**) Dynamic analysis of insulin secretion of nonsorted cells stimulated sequentially by 2 mM glucose, 20 mM glucose, 150 μM tolbutamide.

To further understand the cell composition of stem cell–derived clusters, we performed single-cell RNA sequencing of control and APH-treated cells (Gene Expression Omnibus, GEO: GSE139949). We sequenced 16,739 cells (control = 8091 cells, APH = 8648 cells) on day 27 of differentiation using the MEL1 embryonic stem cell line from both conditions (Supplemental Figure 5A). We first identified 11 cell populations using cells from both control and APH-treated cells, and a large proportion of the cells were endocrine cells (Figure 4A). We then compared these populations corresponding to primary pancreatic human islets (38) (Figure 4B and GEO: GSE114297, GSE139949). Stem cell–derived islet-like clusters contained all endocrine cells identified in primary human islets, including β, α, δ, and pancreatic polypeptide cells. Three stem cell–derived populations (SC-β 2, SC-α, and SC-δ) corresponded most closely to primary human islet β, α, and δ cells (Figure 4B). A total of 69 genes expressed in pancreatic β cells and characteristic of mature cells were enriched in SC-β 2 cluster (including *IAPP*, *SIX2*, *HOPX*, *NEFM*), proinsulin processing and insulin granule exocytosis (*PCSK1*, *CPE*, *PDIA3*, *RAB1A*, *RAB2A*, *RAB3A*, *SCG3*, *VGF*), and metabolism sensing and signaling pathways (*NUCB2*, *PAM*, *G6PC2*, *PDX1*) (Supplemental Figure 5B).

We also identified a group of insulin-expressing cells (SC-β 1) that failed to overlap with the human primary β cell cluster (Figure 4B). Gene Ontology analysis illustrated that genes expressed in SC-β 1 were enriched in the biological process of glycolytic process and molecular function of response to hypoxia, whereas genes in SC-β 2 were highly enriched in the process of hormone transport and secretion (Supplemental Figure 5C). Higher expression of lactate dehydrogenase LDHA, which inhibits mitochondrial activity, and lower expression of key β cell genes were also observed in SC-β 1 (Supplemental Figure 5D). This indicates that SC-β 1 cells are less mature than SC-β 2 cells. Though SC-β 2 cells are closer to human primary β cells than SC-β 1 cells, some of the key β cell markers, including SLC2A1, MIF, and NEUROD1 were expressed at higher levels in SC-β 1 cells (Supplemental Figure 5D and Supplemental Table 7). We also noticed the low detectability of maturation markers, including MAFA, NKX6.1, and HNF1A, at the single-cell level, which did not fully reflect protein levels detected by immunocytochemistry and Western blot (Figure 2E and Figure 4, G and H). Therefore, neither SC-β 1 nor SC-β 2 are identical to pancreatic β cells, pointing out technical challenges in comparing populations by any one set of genes. Other endocrine cells included endocrine cells expressing enterochromaffin cell markers (SC-EC) and a cluster of cells with several hormones (and more similar to pancreatic polypeptide cells, SC-β 2 PP). Nonendocrine cells partially overlap with acinar cells and duct cells in the human islet. Additional cell types, such as endothelial cells or macrophages, were only seen in human islets, while stem cell–derived clusters contained additional nonendocrine cells, including enterochromaffin cells, *FoxJ1*-positive cells, and cells in the cell cycle (Figure 4B). In a comparison with single-cell RNA sequencing data obtained from a published data set (39), we found a high correlation for each cell cluster (Supplemental Figure 6A).

**Figure 5:**
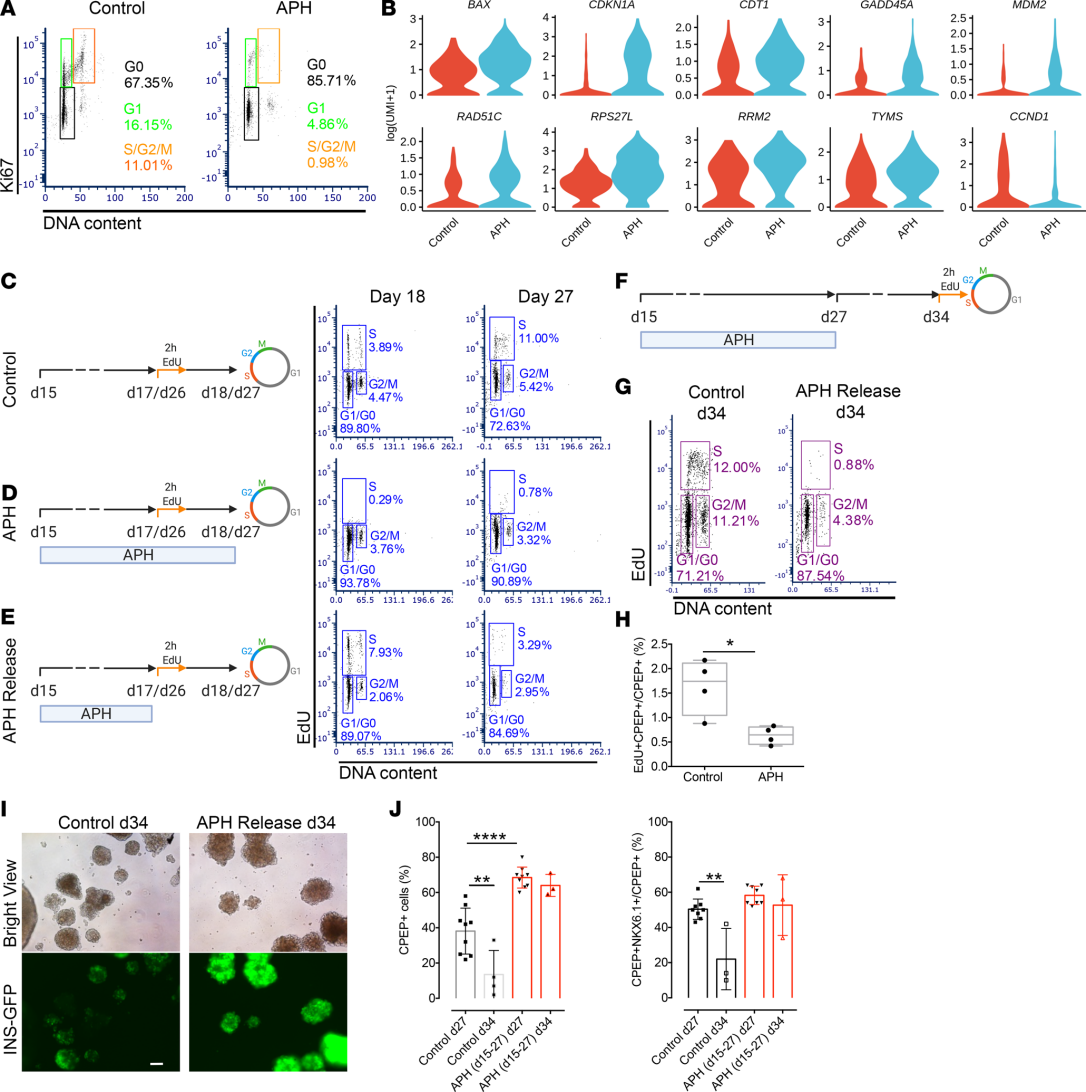
Transient APH treatment reduces growth potential and increases stability of insulin expression by upregulating cell cycle inhibitors. (**A**) Cell cycle profile of untreated and treated cells on day 27 indicated by flow cytometry combined with Hoechst staining for DNA content and KI67 labeling. (**B**) The differential expression of cell cycle genes between control and APH-treated cells using single-cell RNA sequencing. Violin plots show probability density of gene expression of given single cells. (**C**–**E**) Schematic diagrams of cell cycle progression experiments with indicated conditions. (**E**) APH-treated cells were released and labeled with EdU for 2 hours on either day 17 (*n* = 3 in each condition) or 26 (*n* = 4 in each condition), and analyzed 1 day later (day 18 or 27) for cell cycle distribution. Cells without APH treatment (**C**) and unreleased from APH (**D**) were analyzed in parallel. (**F**) A schematic diagram represents the timeline of APH addition, release from cell culture, and cell cycle progression analysis after 2-hour EdU incubation on day 34. Created with BioRender.com (**G**) Cell cycle distribution (*n* = 4) and (**H**) percentage of EdU- and C-peptide–positive cells on day 34 with (*n* = 4) and without APH (*n* = 4) from days 15 to 27. Two-tailed unpaired *t* test *P < 0.05. (**I**) Microscopic pictures of cells on day 34 after releasing cells from APH from day 27 to 34. Scale bar: 100 μm. (**J**) Quantification of C-peptide– and NKX6.1-positive cells and C-peptide–positive cells on days 27 and 34 before and after APH releasing. One-way ANOVA test **P < 0.01; ****P < 0.0001.

We then compared APH-treated and untreated cell populations and quantified their composition. We found a striking reduction in the number of nonendocrine cells in the APH group compared with control (Figure 4C). Furthermore, the proportion of insulin-expressing SC-β 2 cells that are most similar to β cells of primary human islets was significantly increased after APH treatment (Figure 4C). Changes in the number of cells expressing cell cycle genes (*CDKN1A*, *GADD45A*, *BAX*, *MDM2*, *RAD51C*, *RPS27L*, *RRM2*, *CDT1*, and *TYMS*) were also observed. (Detailed data are listed in Figure 5B.) Within SC-β 1 population, the upregulated genes of APH-treated cells included *GNAS*, an important gene for β cell insulin secretory capacity and function (40); *ERO1LB*, a β cell–enriched gene that is involved in insulin processing (41); *ONECUT2*, a transcription factor increased with age in β cells (42); and *TTR*, which has a positive role in glucose-stimulated insulin release (43). *TPI1*, a gene acting in glycolysis, was decreased in APH-treated cells (Figure 4D and Supplemental Table 8). Within SC-β 2 cells, upregulated genes included *IAPP* and *PCP4*, a gene involved in Ca^2+^ binding and signaling (44). The expression of *FEV*, a signature gene expressed in immature β cells (45), was reduced in APH compared with control (Figure 4E and Supplemental Table 8). We also observed that the cell cycle genes (*CITED2* and *CCND1*) were downregulated compared with control in both SC-β 1 and SC-β 2 cells.

To further evaluate β cell markers in a targeted manner, we isolated insulin-positive cells based on GFP expression (Figure 4F) and determined the expression of key β cell genes using Western blot and RT-PCR. We found that protein levels of PDX1, NKX6.1, v-maf musculoaponeurotic fibrosarcoma oncogene homolog A (MAFA), and Proinsulin were all upregulated in GFP-positive cells isolated from APH-treated clusters compared with GFP-positive cells in control clusters (Figure 4, G and H). The higher protein levels correlated with increased transcription levels (Supplemental Figure 7A and Supplemental Table 3). In addition, APH-treated cells produced higher newly synthesized proinsulin normalized to total protein synthesis (Figure 4I). These data show that APH-induced cell cycle arrest promotes a gene expression program characteristic of more mature cells.

To examine the effect of APH on the functionality of insulin-producing cells, static and dynamic glucose-stimulated C-peptide secretion were evaluated. In response to elevated glucose, C-peptide secretion was increased 2- to 6-fold, with an average of 3-fold in a static assay, significantly more than in untreated controls (Figure 4J). The levels of basal insulin secreted by APH-treated cells were comparable with those in control (Figure 4K). Dynamic perifusion demonstrated that APH-treated insulin-producing cells showed a response to high glucose and to tolbutamide comparable to controls (*n* = 3) (Figure 4L).

Therefore, cell cycle arrest triggered by APH reduces the proportion of nonendocrine cells and has no adverse effects on the functional properties of differentiated cells.

### Transient treatment with APH limits proliferation potential and stabilizes insulin expression by upregulating cell cycle inhibitors in vitro.

To determine whether APH has lasting effects on cell cycle progression, we examined the expression of cell cycle markers at the end of treatment on day 27. As shown in Figure 5A, 86.4% ± 0.9% (*n* = 3) of cells treated with APH versus 74.3% ± 6.5% (*n* = 3) of cells in control conditions were found to exit the cell cycle to G_0_. A total of 14.4% ± 11.2% (*n* = 3) of control cells were in the cell cycle, as indicated by the expression of KI67 and DNA content. About 2.9% ± 1.8% (*n* = 3) of APH-treated cells expressed KI67 in G_1_, and very few of the cells were in S phase (Figure 5A). We also examined the cell cycle gene expression in insulin-expressing cells by isolating GFP-positive cells from cell clusters. We found that the expression of CDK inhibitor 1A (*CDKN1A*, a cell cycle progression inhibitor) was upregulated, and the expression of cyclin D1 (*CCND1*) and *CDK4* (both involved in G_1_ phase progression) was downregulated in APH-treated insulin-positive cells (Figure 5B and Supplemental Figure 7B). These results demonstrate that APH promotes G_1_/G_0_ arrest and induces S and G_2_/M arrest in pancreatic endocrine progenitors.

A subset of cells appeared competent of DNA replication after APH treatment. In KI67-positive cells, single-cell RNA sequencing showed upregulation of genes involved in the P53 signaling pathway (*CDKN1A*, *GADD45A*, *BAX*, *MDM2*, *RPS27L*, *RRM2*) in APH-treated cells. These genes mediate G_1_ cell cycle arrest or respond to difficulties in DNA replication progression. *CDKN1A* and *GADD45A* are able to arrest cells either in G_1_/S or G_2_/M (46). Chromatin licensing and DNA replication factor 1 (*CDT1*), a replication licensing gene (stable in G_1_ and degraded in S phase), was also upregulated, indicating that cells were arrested in late G_1_ (Figure 5B). *CDT1* upregulation appears to be a compensatory response to enable S phase progression and attempt to rescue S phase in the presence of APH.

To explore the consequences of transient APH treatment on proliferation potential, we exposed pancreatic progenitors to APH and released them at different time points (Figure 5, C–E). On day 17, 2 days after APH treatment, APH was removed from culture, and cells were incubated with EdU for 2 hours and collected the next day (day 18). As shown in Figure 5D, 93.3% ± 1.1% (*n* = 3) of cells were arrested in G_1_/G_0_, and very few cells, 0.4% ± 0.4% (*n* = 3), went through S phase when APH was present. Upon removal of APH, 6.0% ± 1.8% (*n* = 3) of cells resumed proliferation, as indicated by EdU staining, more than in control (3.2% ± 0.8%) (*n* = 3) (Figure 5E). When cells were treated for 12 days and released from APH on day 26, 3.4% ± 1.1% (*n* = 4) of cells were EdU positive on day 1 after releasing (day 27), while in controls 7.9% ± 3.7% (*n* = 4) were proliferating (Figure 5, C and E). Therefore, while short-term exposure induced enrichment at the G_1_/S phase transition and S phase could resume, long-term exposure increased G_0_ arrest, while approximately 3% were in G_1_ (Figure 5A) and capable of reentering S phase (Figure 5E).

To test the stability of G_0_/G_1_ arrest, we continued culturing cells after release from APH for 7 days till day 34 and labeled with EdU on day 34 for 2 hours (Figure 5F). The percentage of EdU-positive cells was substantially reduced to 2.1% ± 1.2% (*n* = 4), whereas control cells continued proliferating at a rate of 12.0% ± 3.4% (*n* = 4) (Figure 5G). The percentage of proliferating C-peptide–positive cells was also reduced from 1.6% ± 0.6% (*n* = 4) in controls to 0.6% ± 0.2% (*n* = 4) in APH-treated cells (Figure 5H). Thus, growth potential was greatly reduced across all cell types, when replication fork speed was slowed by APH during 12 days of differentiation, and the vast majority of cells had entered a stable G_0_ state.

To explore if the lasting effect of APH on cell cycle progression contributes to maintain β cell identity, insulin-producing cells were cultured for an additional 7 days till day 34 upon removal of inhibitors on day 27. In untreated control cells and CDK4i-treated cells, the insulin-GFP expression was lost while it remained high in APH-, Cis-, and Eto-treated cells on day 34 (7 days after release) (Figure 5I and Supplemental Figure 7C). In addition, the percentage of C-peptide and NKX6.1 double-positive cells and the total of C-peptide–positive cells were still high on day 34 in cells treated with APH, Cis, and Eto, respectively, whereas the percentage of C-peptide–positive cells was significantly reduced in the control and the CDK4i groups (Figure 5J and Supplemental Figure 7D). Cells treated with low dose of APH (0.1 μM) were comparable to untreated controls in cell composition (Supplemental Figure 7E). These data show that transient treatment with APH (≥0.25 μM) (and other inhibitors of DNA replication) results in more stable β cell identity and that the stability of insulin-expressing endocrine cells subsequently becomes independent of the compounds.

### APH-treated cells show reduced growth potential in vivo in a dose-dependent manner.

**Figure 6:**
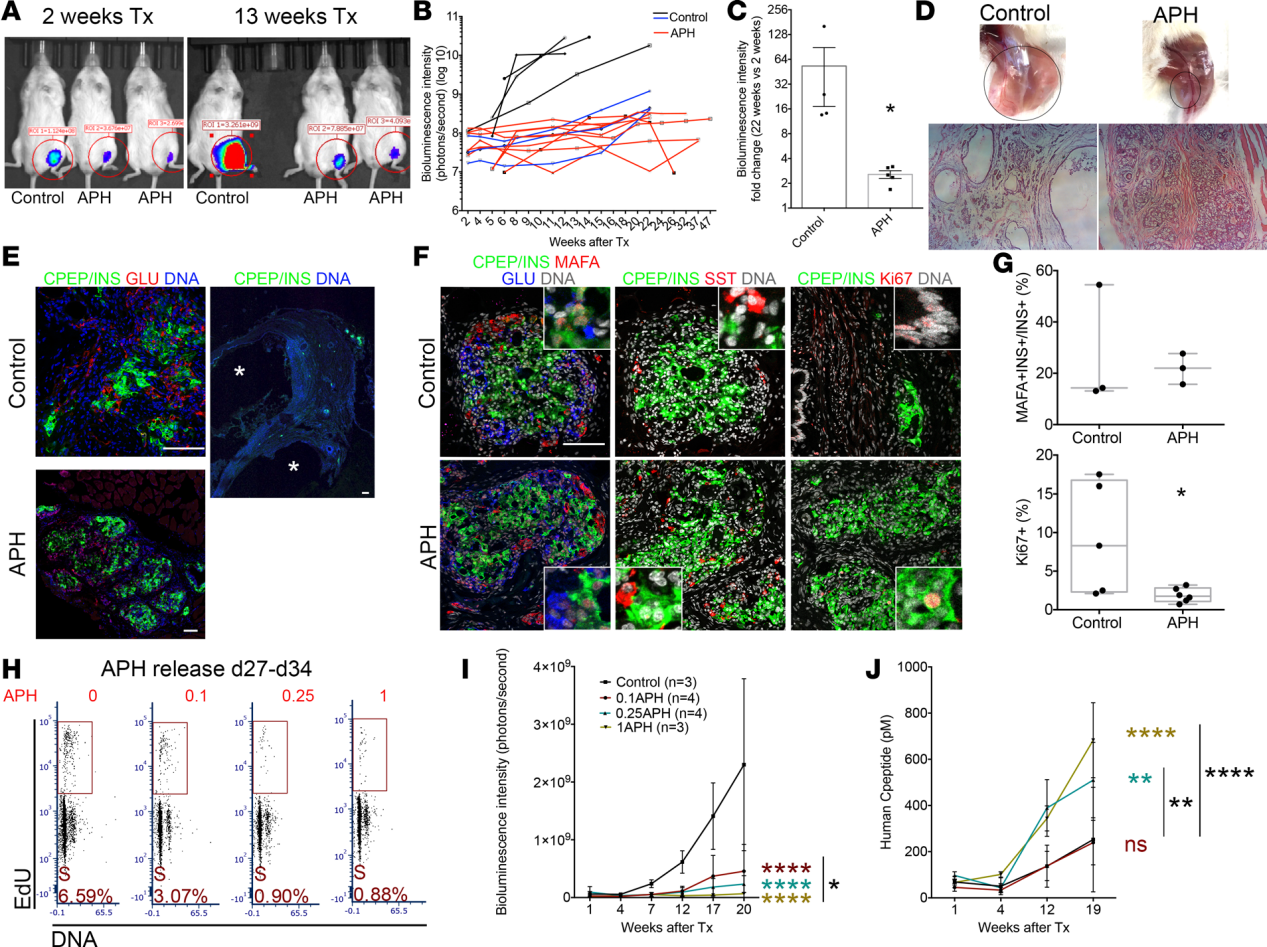
Reducing replication fork speed and S phase entry establishes cell-intrinsic limitations in cell proliferation. (**A**) In vivo imaging of mice transplanted with control and APH cells. Tx, transplantation. (**B**) Growth of grafted cells in mice after transplantation quantified by the bioluminescence intensity. Differentiation efficiency and graft growth in controls are variable but consistent in APH treatment. Black and blue lines show mice transplanted with control clusters (black, approximately 20% differentiation efficiency, *n* = 4; blue, >60%, *n* = 3). (**C**) Fold change of graft growth in control and APH mice. *Mann-Whitney *U* test with *P* < 0.05. (**D**) Grafts in the legs of mice transplanted with equal number of cells in control and APH group at 20 weeks (black circles, graft location). (**E** and **F**) Cell composition determined by immunostaining in mice transplanted with control and APH cells. *cysts. (Pictures with white line: close-ups.) Scale bar: 100 μm. (**G**) Quantification of MAFA- and INS-positive cells (control: 2 grafts from 2 mice from 1 batch of differentiation, INS^+^ cells counted from 3 sections; APH: 2 grafts from 2 mice from 2 batches of differentiation, INS^+^ cells counted from 3 sections) and KI67-positive cells (control: 2 grafts from 2 mice from 1 batch of differentiation, cells counted from 5 sections; APH: 3 grafts from 3 mice from 2 batches of differentiation, cells counted from 6 sections) (Supplemental Table 6). Two-tailed paired *t* test *P < 0.05. (**H**) Representative day 34 cell cycle distribution with day 15–day 27 APH pretreatment (*n* = 2). (**I**) Cell growth in mice after transplantation of cells pretreated with APH measured by bioluminescence intensity. Two-way ANOVA *P < 0.05 (0.1APH vs. 1APH); ****P < 0.0001 (0.1, 0.25, 1APH vs. control). (**J**) Human C-peptide secretion in mice transplanted with APH-treated cells. Two-way ANOVA **P < 0.05 (0.1APH vs. 0.25APH in black; 0.25APH vs. control in color ); ****P < 0.0001 (0.1APH vs. 1APH in black; 1APH vs. control in color).

To determine long-term effects of cell cycle arrest and reduced replication fork speed on growth potential, we removed APH on day 27 and monitored the graft growth after transplantation in vivo. Control and APH-treated cells were prepared from 3 independent differentiation experiments using a MEL1 cell line. Within each experiment, the same number of cells were transplanted in APH and control groups. MEL1 cell line was targeted with a luciferase reporter under the control of the *GAPDH* promoter, which allows us to monitor cell growth in vivo (Supplemental Table 1). APH was removed permanently on day 27 before transplantation. Graft growth was evaluated by monitoring a luciferase reporter using in vivo imaging. After 2 weeks of transplantation, the graft size of APH mice was small, while controls grafted with the same number of cells were modestly larger (Figure 6A). Eleven weeks later, 4/7 control mice displayed large growths, whereas none of the mice transplanted with APH-treated cells did (9/9) (Figure 6A). The different growth trend of grafted cells between control and APH-treated cells was evident in the bioluminescence intensity (Figure 6B). At 22 weeks of engraftment, the size of graft in the APH group was on average 2.6-fold larger than that at 2 weeks, while the size of the control group increased on average by 53-fold (Figure 6C). Graft growth occurred in controls even from cultures with very high differentiation efficiency (>60%). Even in mice with the smallest growths of control cells, cystic structures still formed in 3/3 mice (Figure 6B, indicated by blue lines). No cysts were observed in mice grafted with 1 μM APH-treated cells in 9/9 mice (Figure 6D). Grafts were isolated from mice for examination. Both control graft and APH graft were composed of islet-like structures and showed monohormonal cells positive for insulin, for glucagon, or for somatostatin (Figure 6, E and F). Approximately 25% of insulin-expressing cells stained positive for MAFA, and no difference in the proportion of insulin- and MAFA-positive cells was observed between control and APH (Figure 6, F and G). In mice grafted with untreated control cells, the graft contained groups of C-peptide–positive cells but developed several large cystic structures after 9 months of transplantation (Figure 6E) with approximately 10% of cells positive for KI67 (Figure 6, F and G). Grafts derived from APH-treated cells had approximately 2% KI67-positive cells (Figure 6, F and G), demonstrating that APH had altered growth potential and did not damage cells without any proliferative capacity. We also transplanted iPSC-derived clusters into NOD.Cg-*Prkdc^scid^* *Il2rg^tm1Wjl^*/SzJ (NSG) mice to determine if growth control by APH also applied to iPSCs. No cysts were observed in mice grafted with APH-treated iPSC-derived clusters (0/3), whereas cysts were formed in mice transplanted with nontreated cells (2/2) (Supplemental Figure 8A).

APH reduces replication fork speed in a dose-dependent manner (Figure 1, B and C). To determine if growth potential correlates with the degree of reduction in replication fork speed, we released cells on day 27 from different concentrations of APH and analyzed them using EdU staining 1 week later. Without APH pretreatment, cells kept proliferating on day 34. In contrast, cells pretreated with APH showed a reduced proliferation rate proportional to replication fork progression (Figure 6H and Figure 1B). We then transplanted cells pretreated with 0.1 μM, 0.25 μM, and 1 μM of APH into mice and monitored graft growth with bioluminescence intensity for 17 weeks. A comparable number of cells and clusters were engrafted. Accordingly, comparable bioluminescence intensity of grafted cells was observed among all 4 groups at 1 week of transplantation. A difference in graft size was apparent at 7 weeks of transplantation and became statistically significant at 12 weeks and 17 weeks posttransplantation (Figure 6I). APH treatment resulted in a dose-dependent reduction in growth potential: grafts were smallest for the highest concentration of APH and intermediate to controls for the lowest concentration (Figure 6I).

While the difference in graft size to controls was obvious at all tested APH concentrations, the levels of human C-peptide secretion in mice transplanted with control cells and mice transplanted with low concentration (0.1 μM) of APH-treated cells remained similar, consistent with cell composition (Figure 6J). Mice transplanted with 0.25 μM and 1 μM APH-treated cells secreted higher levels of human C-peptide starting from 4 weeks after transplantation compared with control and low APH (Figure 6J). In contrast to the higher concentrations of APH, low concentrations selectively reduced growth potential but had no effect on cell composition. Thus, replication fork speed altered by APH affects control growth potential in a dose-dependent manner and prevents teratomas and cystic growth after transplantation.

### APH-treated cells secrete C-peptide and efficiently protect mice from diabetes.

**Figure 7:**
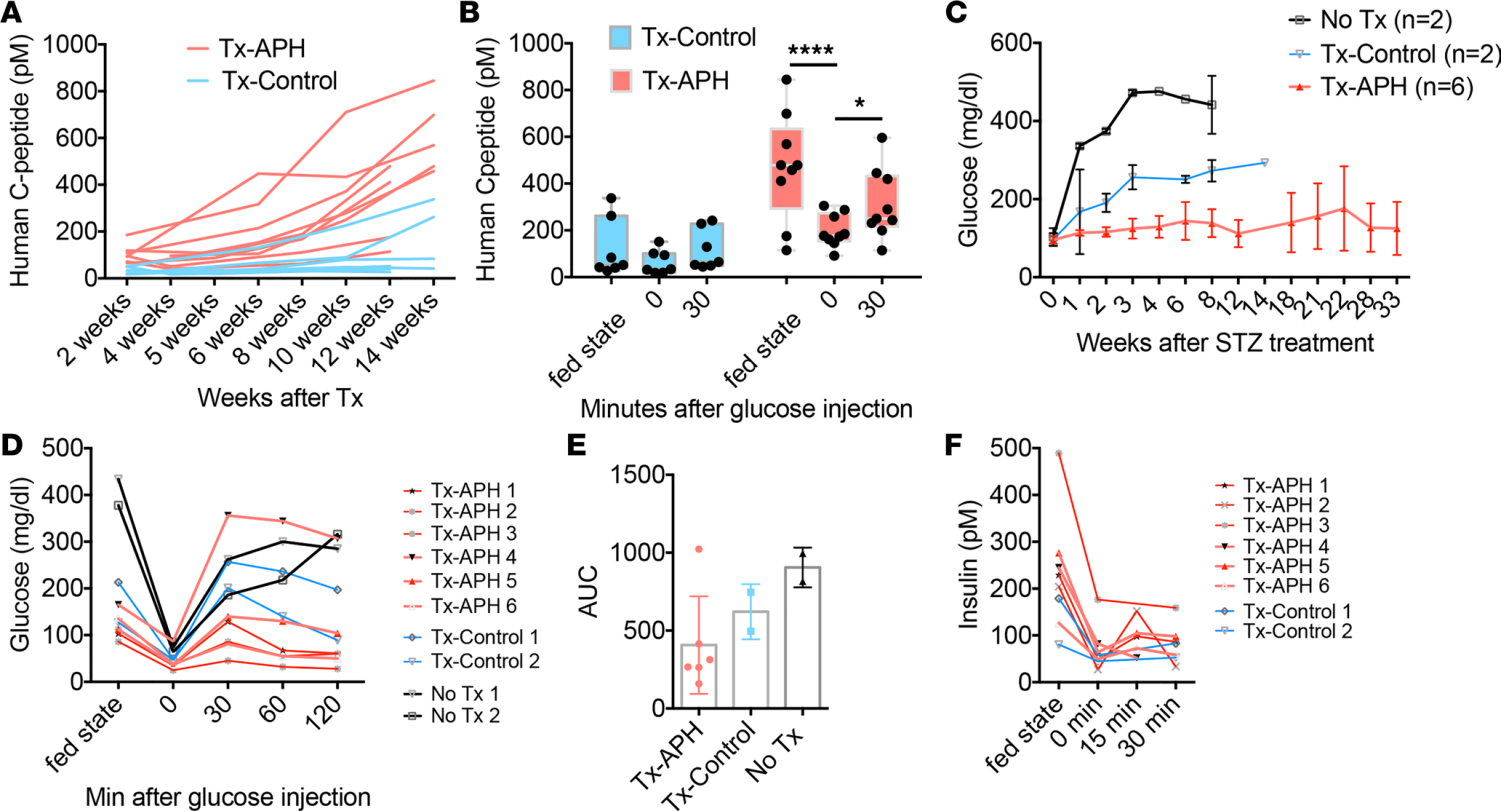
Mice transplanted with cells pretreated with APH secrete high human C-peptide and are protected from induced diabetes. (**A**) Human C-peptide serum concentration in mice at different time points after transplantation with cells treated with APH (APH) and control cells (MEL1) at fed state. (**B**) Human C-peptide serum concentration in mice at 12–14 weeks after transplantation with APH cells (APH) and control cells (MEL1) at fed state, fasting, and 30 minutes after glucose injection. Two-way ANOVA *P < 0.05; ****P < 0.0001. (**C**) Blood glucose levels of STZ-treated mice without transplantation (No Tx) (*P* = 2), transplanted with control cells (Tx-Control) (*n* = 2), and with APH-treated cells (Tx-APH) (*n* = 6). Blood glucose levels of Tx-Control and No Tx mice were monitored until persistent hyperglycemia required euthanasia according to animal protocol. (**D**) Glucose tolerance test of STZ-treated mice in fed state, fasting state, and 15–120 minutes after glucose injection. Intraperitoneal glucose tolerance test (IPGTT) was performed on day 14 after STZ treatment. (**E**) Area under the curve of glucose tolerance test was calculated to compare among Tx-Control, Tx-APH, and No Tx. (**F**) Serum human insulin concentrations of STZ-treated mice transplanted with APH-treated cells at fed state, fasting, and 30 minutes after glucose injection.

To test in vivo function of APH-treated islet-like clusters, we monitored C-peptide and blood glucose levels. After transplantation in immunodeficient mice, mice transplanted with APH-treated cells trended to higher human C-peptide starting from 2 weeks after engraftment, compared with controls (Figure 7A). The increase was statistically significant at 6 weeks after transplantation (Figure 7A and Supplemental Figure 8, B and C). Secretion of human C-peptide in mice was downregulated when mice were fasted and increased after glucose injection (Figure 7B), indicating the engrafted insulin-producing cells were able to respond to changes in blood glucose levels.

The ability of APH-treated cells to protect mice from diabetes was determined after eliminating endogenous mouse β cells with streptozotocin (STZ). STZ ablates mouse β cells but is not toxic to human β cells at the concentrations used (29). After 15 weeks of transplantation, mice were treated with STZ, blood glucose levels were monitored, and grafted insulin-producing cells were challenged with high glucose to check their function. Successful ablation of mouse β cells with STZ was demonstrated by mouse C-peptide ELISA (Supplemental Figure 8D) and was previously also shown to result in the loss of C-peptide staining in the mouse pancreas (29). Blood glucose levels remained in the normal range in 5 out of 6 mice transplanted with APH-pretreated cells (Figure 7C). Five out of 6 mice were tolerant to glucose and normalized blood glucose levels within 60 minutes of glucose injection (Figure 7, D and E). Secretion of human C-peptide and insulin decreased after fasting and increased after glucose injection (Figure 7F and Supplemental Figure 8E). Therefore, APH-treated cells control graft growth while protecting mice from diabetes more efficiently than nontreated cells.

## Discussion

Modulation of signaling pathways has been used successfully to differentiate pluripotent stem cells to insulin-producing cells (47–50). In this study, we tested the duplication of the genome as a developmentally relevant target to induce the differentiation of stem cell–derived endocrine cells and to establish limitations in cell proliferation. Unlike the modulation of signaling pathways that can have complex effects on both gene expression and cell cycle progression, the use of APH is highly specific in targeting the duplication of the genome by inhibiting DNA polymerase in a dose-dependent manner. APH affects the progression from G_1_ to S phase, while also affecting DNA replication completion, in particular at common fragile sites (28). Common fragile sites replicate late in S phase, and are prone to incomplete replication, a property that is enhanced through addition of APH. A number of compounds tested here arrested pancreatic progenitors in G_1_, but only DNA replication inhibitors APH, cisplatin, and etoposide substantially increased the production of insulin-producing cells. The key difference of DNA replication inhibitors from other cell cycle inhibitors is the compromised cell cycle progression from S to G_1_ phase. In the presence of DNA replication inhibitors, the progression through G_2_/M phase was impaired and arrested during the progression to G_1_. A recent study demonstrated that the inhibition of YAP in the Hippo signaling pathway increases the differentiation of pancreatic endocrine cells from pancreatic progenitors. In contrast, inhibition of cell cycle progression with roscovitine, a CDK inhibitor, did not achieve the same effect as YAP (51). Our studies point out that G_1_ arrest by CDK inhibition alone is not sufficient to promote β cell differentiation but is effective when combined with compromised S phase progression. Whether YAP mediates its effect on β cell differentiation by affecting origin activity or replication fork progression is not currently known.

Inhibition of DNA replication reduced variation in differentiation efficiency in different experiments with the same cell line, as well as with different cell lines. Stem cell lines that were previously demonstrated as differentiation incompetent (29) also showed improved differentiation efficiency. Single-cell RNA sequencing data revealed that the number of mature β cells (SC-β 2) that are transcriptionally more similar to human primary β cells was increased. APH-treated cells showed upregulated expression of genes in metabolic signaling and insulin processing and release and downregulated expression of genes in cell cycle progression and glycolysis. Furthermore, APH treatment decreased the number of nonendocrine cells, both in comparison with our controls, as well as compared with another study (39) (Supplemental Figure 6A). Though the resulting cells are closer, they are not identical to pancreatic β cells in gene expression program and function. Additional adaptations to increase glucose responses may result in further functional improvements (50). Taken together, APH treatment increases the robustness of the differentiation protocol.

The study of DNA replication adds a new perspective to the existing literature on cell cycle progression in the pancreatic lineage. Pancreatic endocrine differentiation is associated with cell cycle exit. A recent study demonstrated that overexpression of islet cell enriched miRNA repressed the expression of cell cycle regulators at the transcription level (52). Our studies demonstrate a role of reduced S phase competence in the commitment of pancreatic progenitors to the endocrine lineage during differentiation from pluripotent stem cells. Reduced S phase competence is established naturally in pancreatic differentiation, though with slower developmental kinetics than induced by APH or antineoplastic agents: human β cells are not only arrested in G_1_; they are also compromised in their ability to complete S phase as shown by forced S phase entry of β cells, which can induce apoptosis (7). In the brain, replication incompetence is even greater: neuronal cells that are forced to reenter a cell cycle through inhibition of Rb will die and degenerate rather than divide and grow (6, 53). Therefore, mechanisms that impair S phase entry and S phase completion may be an important and fundamental principle of terminal differentiation in several organs, in particular in the brain, in muscle cells, and in the pancreas. S phase completion and S phase reentry are functionally linked, as the decision to enter quiescence in G_1_ is determined by the previous S phase (37, 54).

Other studies have focused on developmental signals and the activity of transcription factors, such as *NGN3*, in understanding growth and terminal differentiation of the pancreas (55). However, how *NGN3* and other transcription factors affect cell cycle progression and DNA replication remains to be further investigated. In our study, we show that reducing DNA replication progression is sufficient to promote pancreatic endocrine differentiation from progenitors and increase *NGN3* expression. Transcription factors may modulate S phase entry and progression not only through the expression of gene products, but also by altering origin activity.

Our findings are relevant to defining growth potential of cell replacement products for the treatment of diabetes or other conditions. In this and previous studies, a proliferative nonendocrine population in untreated controls can contribute to growths after transplantation (29, 56). Several strategies have been developed to reduce the risk of such outcomes. A flow cytometry–based method can be used to purify stem cell–derived insulin-producing cells labeled with a fluorescent reporter to prevent teratomas (49) and with an identified β cell surface marker CD49a (39). Furthermore, genetically engineering a suicide gene in a stem cell line can efficiently kill non–insulin-producing cells upon drug administration at the end of differentiation (57). The rationale for the methods developed here is based on the biology of the mature β cell: cell cycle exit and a compromised ability to undergo S phase. Through the use of small molecules promoting cell cycle exit, growth after transplantation is controlled and teratomas and cysts are avoided. This method does not require transgenes or flow cytometry and is readily transferable between different cell lines and cell types. Remarkably, APH-treated cells showed consistent graft size up to 1 year after transplantation. Therefore, we show that growth limitations can be modulated by specific and transient interference with DNA replication. Though we do not know how closely APH mimics developmental processes, it is of interest to note that neurons exiting the cell cycle incur frequent breaks and copy number changes in late replicating regions (58, 59). These same sites are targeted by low doses of APH, including the concentrations used here (28). Therefore, our studies suggest a physiological role of fragile sites in mediating limitations of growth.

In conclusion, we demonstrate an active role of limitations in DNA replication in the stable commitment to an endocrine cell fate during differentiation of human pluripotent stem cells. The study of differentiation in an in vitro stem cell system is limited by the non-native environment, and the insight gained here may not be directly applicable to in vivo development. However, human pluripotent stem cells have emerged as an increasingly powerful model to understand the human genome in development and disease (60). Understanding the molecular mechanisms that limit replication potential is central to our understanding of development and for the use of pluripotent stem cells in regenerative medicine.

## Methods

### Human pluripotent stem cell culture.

Human pluripotent stem cells were cultured and maintained on feeder-free plates with StemFlex Medium (catalog A3349401, Thermo Fisher Scientific) as described (61). Four cell lines were involved in this study as shown in Supplemental Table 1: MEL1 is human embryonic stem cell line (62); 1023A is a human iPSC line derived by reprogramming a skin fibroblast biopsied from a healthy control; 1018E is a human iPSC line reprogrammed from a skin fibroblast biopsied from a female type 1 diabetes patient (29); and 1159 is a human iPSC line reprogrammed from a skin fibroblast biopsied from a female healthy control (29) (Supplemental Figure 10). MEL1 cell line was used to generate data in Figures 1–7. Data derived with other cell lines are shown in the supplemental figures.

### Differentiation to insulin-producing cells from human pluripotent stem cells.

Insulin-producing cells were differentiated from human pluripotent stem cell lines using published protocol with modifications (61). Refer to the Supplemental Methods for additional details.

### Treatment with DNA replication inhibitors and cell cycle inhibitors.

The DNA polymerase inhibitor APH (catalog A0781, MilliporeSigma) was added with indicated concentrations at specified time points from day 15 to 27 or as shown in figures. Other compounds used to interfere with cell cycle progression are listed in Supplemental Table 2. The concentrations of compounds were determined based on the survival of cells.

### Replication progression analysis.

Cell clusters on day 15 were incubated sequentially with 25 μM IdU and 25 μM CIdU for 30 minutes each in the presence of APH with indicated concentrations. Cell clusters were collected and dissociated into single cells, and DNA fibers were stretched and stained for IdU and CIdU as described in Terret et al. (63). A total of 1 μm DNA fibers was considered 2.6 kb as described (64). Refer to the Supplemental Methods for additional details.

### Cell cycle progression analysis.

Cell clusters at day 17 or day 26 in the indicated conditions were incubated with EdU for 2 hours and then washed twice to remove EdU. Cell clusters were continuously cultured in the medium with or without indicated compounds, collected at the next day, and dissociated for flow cytometric analysis. EdU was stained by Click-iT EdU Alexa Fluor 555 Imaging Kit (catalog C10338, Thermo Fisher Scientific) followed by Hoechst 33342 staining. Flow cytometry was performed to determine the number of cells in each phase of the cell cycle. Refer to the Supplemental Methods for additional details.

### Immunocytochemistry.

Clusters at day 27 were collected and fixed with 4% paraformaldehyde (PFA) at room temperature for 10 minutes. Grafts were taken from the mice and fixed with 4% PFA at room temperature for 1 hour. The following steps were performed according to the published method (61). Primary antibodies are listed in Supplemental Table 4, and secondary antibodies are listed in Supplemental Table 5. Pictures were taken with an OLYMPUS IX73 fluorescent microscope or ZEISS LSM 710 confocal microscope.

### Single-cell RNA sequencing and read mapping.

Single cells were suspended in PBS + 0.04% BSA. Totalseq-A anti-human hashtag antibodies (catalog 394601, 394603, 394605, 394607, 394609, 394611, 394613, 394615, BioLegend) were used for cell hashing. Each sample was individually stained with one of the hashtag antibodies and washed 3 times. Eight samples were pooled at equal concentrations, and the pool was loaded into the Chromium instrument (10x Genomics) at 32,000 cells per lane. Single-cell RNA sequencing libraries were prepared using Chromium Single Cell 3′ Reagent Kits v2 (10x Genomics). Hashtag libraries were generated as described previously (65). Sequencing was performed on Illumina NextSeq500. Sequence alignment and expression quantification were performed using Cell Ranger Single-Cell Software Suite (10x Genomics, v2). Reads were aligned to the B37.3 Human Genome assembly and UCSC gene model. Refer to the Supplemental Methods for additional details.

### Gene ontology analysis.

The functional enrichment analysis was performed using g:Profiler (version e97_eg44_p13_d22abce) with g:SCS multiple-testing correction method applying significance threshold of 0.01 (66).

### Dynamic glucose-stimulated insulin secretion.

Microfluidic-based perifusion system was used to determine glucose-stimulated insulin secretion. This experiment was conducted according to previously described methods (67, 68). Refer to the Supplemental Methods for additional details.

### Western blotting.

The sorted GFP-positive and -negative cells with and without APH treatment were lysed with RIPA buffer. Refer to the Supplemental Methods for additional details.

### Transplantation and in vivo assay.

Male 8- to 10-week-old immunocompromised mice (NOD.Cg-*Prkdc^scid^* *Il2rg^tm1Wjl^*/SzJ [NSG] from The Jackson Laboratory, catalog 005557) were used for transplantation. For intra–leg muscle transplantation, approximately 2 million cells were collected and transferred to a tube with 50 μL Matrigel. They were injected in the leg muscle with a 21-gauge needle. Refer to the Supplemental Methods for additional details.

### Data availability.

Single-cell transcriptome data of stem cell–derived cell clusters (control vs. APH) were deposited in GEO: accession GSE139949 (note: WT = Control). Single-cell sequencing data of human primary islets were deposited in GEO: accession GSE114297 (38).

### Statistics.

Statistical tests performed for specific data sets are described in the corresponding figure legends. Data were analyzed as indicated in figure legends by a 2-tailed unpaired or paired *t* test and by 1-way or 2-way ANOVA followed by Tukey’s multiple-comparison test (GraphPad Prism 6, GraphPad Software, Inc.) and plotted as mean ± standard deviation. The differences observed were considered statistically significant at the 5% level and were displayed on figures as follows: *P < 0.05, **P < 0.01, ***P < 0.001, ****P < 0.0001.

### Study approval.

Derivation of the lines has been previously described and was performed after receiving informed consent and using protocols reviewed and approved by the Columbia University Institutional Review Board and the Columbia University Embryonic Stem Cell Committee. All animal protocols were approved by the Institutional Animal Care and Use Committee of Columbia University.

## Author contributions

LS conceived and designed the study, performed experiments, analyzed data, and wrote the manuscript with input from all authors; Y Xin performed single-cell RNA sequencing analysis; GD performed Western blot and RT-PCR; DG performed DNA fiber analysis; QD performed 53BP1 and γH2AX foci analysis; LH and PA performed proinsulin biosynthesis analysis; YH, YW, and Y Xing performed microfluidic perifusion and data analysis; MVZ performed live imaging; JF assisted with GSIS, in vivo imaging, and ipGTT; DB assisted with drug testing; QS performed and optimized single-cell RNA sequencing analysis; JK and SK dissociated β cell clusters for single-cell RNA sequencing; RSG coordinated human subject research; SK discussed RNA sequencing results; and DE conceived the study and contributed to experimental design, data interpretation, and manuscript writing. DE, RSG, PA, SK, FB, and JO provided supervision.

## Supplementary Material

Supplemental data

Supplemental Video 1

Supplemental Video 2

Supplemental Video 3

Supplemental Video 4

Supplemental Video 5

Supplemental Video 6

## Version 1 02/02/2021

In-Press Preview

## Version 2 03/08/2021

Electronic publication
