# Supplementary material for: Reduced replication fork speed promotes pancreatic endocrine differentiation and controls graft size
Source: JCI Insight. 2021 Mar 8;6(5):e141553. doi: 10.1172/jci.insight.141553 (PMC8022502; doi:10.1172/jci.insight.141553)
Supplement: Supplemental data [file jciinsight-6-141553-s008.pdf]

Supplemental Materials and Methods:

Differentiation to insulin-producing cells from human pluripotent stem cells

Insulin-producing cells differentiated from human pluripotent stem cell lines using published protocol with modifications (Sui et al., 2018b). Briefly, cells were cultured for 4 days using STEMdiff™ Definitive Endoderm Differentiation Kit (Cat. No. 05110, STEMCELL Technologies) for definitive endoderm induction. Media was changed to RPMI 1640 plus GlutaMax (Cat. No. 61870-127, Life Technology) + 1% (v/v) Penicillin-Streptomycin (PS) (Cat. No. 15070-063, Thermo Fisher Scientific) + 1% (v/v) B-27 Serum-Free Supplement (50x) (Cat. No. 17504044, Life Technology) + 50 ng/ml FGF7 (Cat. No. 251-KG, R&D System) for 2 days. From day 6 to day 8, media was changed to DMEM plus GlutaMax (DMEM) (Cat. No. 10569-044, Life Technology) with 1% (v/v) PS + 1% (v/v) B-27 + 0.25 μM KAAD-Cyclopamine (Cat. No. 04-0028, Stemgent) + 2 μM Retinoic acid (Cat. No. 04-0021, Stemgent) + 0.25 μM LDN193189 (Cat. No. 04-0074, Stemgent). To generate pancreatic progenitors, cells were refreshed in DMEM + 1% (v/v) PS + 1% (v/v) B-27 + 50 ng/ml EGF (Cat. No. 236-EG, R&D System) + 25 ng/ml FGF7 for 4 days. At day12, cells were dissociated into single cells and clustered in AggreWell 400 6-well plates (Cat. No. 34425, STEMCELL Technologies) with DMEM + 1% (v/v) PS + 1% (v/v) B-27 + 1 μM ALK5 inhibitor (Stemgent, cat. no. 04-0015) + 10 μg/ml Heparin (Sigma-Aldrich, cat. no. H3149) + 25 ng/ml FGF7 + 10 μM Y-27632, ROCK inhibitor. At day 13, newly formed clusters were transferred into Low-attachment 6-well plates (Cat. No. 07-200-601, Thermo Fisher Scientific) and cultured with RPMI 1640 plus GlutaMAX + 1% (v/v) PS + 1% (v/v) B-27 + 1 μM thyroid hormone (T3) (Cat. No. T6397, Sigma) + 10 μM ALK5 inhibitor + 0.1 μM LDN193189 + 10 μg/ml heparin +100 nM gamma-secretase inhibitor (DBZ) (EMD Millipore, cat. no. 565789) + 10 μM Y-27632, ROCK inhibitor for 7 days. From day 20 to day 27, clusters were cultured in

RPMI 1640 plus GlutaMAX + 1% (v/v) PS + 1% (v/v) B-27 + 10% (v/v) fetal bovine serum (Atlanta Biologicals, cat. no. S11150) + 10 μM zinc sulfate + 10 μg/ml heparin + 10 μM Y-27632.

Replication progression analysis

Briefly, drops of cell suspension in PBS were spotted on glass slides and lysed with 0.5% SDS, 200mM Tris pH 7.4, 50mM EDTA. Fibers were then stretched by tilting the slides at a 10-15° angle and fixed with methanol: acetic acid = 3:1. Staining was performed with the following antibodies- anti-BrdU/CldU (Biorad # OBT0030) and anti-BrdU/IdU (BD # 347580). Stained DNA fibers were imaged with the 100x objective of an Olympus inverted fluorescence microscope IX73 and measured with Olympus cellSens analysis software.

Static glucose stimulated C-peptide secretion

Krebs Ringer buffer (KRB) was prepared by addition of 129 mM NaCl, 4.8 mM KCl, 2.5 mM $CaCl_2$, 1.2 mM $MgSO_4$, 1 mM Na_2HPO_4, 1.2 mM KH_2PO_4, 5 mM $NaHCO_3$, 10 mM HEPES and 0.1% BSA in deionized water and was sterilized with a 0.22 μm filter. 2 mM and 20 mM glucose solution were prepared in KRB for low glucose and high glucose challenge of sc-beta cell clusters. 10-20 sc-beta cell clusters (~$5x10^5$ cells) were collected from control and APH treated conditions and pre-incubated in 500 μl low glucose solution for 1 hour. Clusters were then washed once with low glucose solution and subsequently incubated in 200 μl of low glucose and then high glucose solution for 30 min. 130 μl supernatant from each condition was collected. Supernatants containing C-peptide, secreted in low and high glucose conditions were processed using Mercodia Ultrasensitive Human C-peptide ELISA kit (Cat: 10-1141-01, Mercodia). Fold changes of C-peptide secretion before and after glucose stimulation were calculated.

Dynamic glucose stimulated insulin secretion

100 clusters from each group were perfused sequentially with KRB containing 2 mM glucose (10 min), 20 mM glucose (30 min), 150 μM Tolbutamide (20 min) and 2 mM glucose (20 min). Perifusate samples were collected at the outlet at flow rate of 200 μl/min and every 2 min samples were used to determine insulin concentration by Mercodia Ultrasensitive Human Insulin ELISA kit.

Flow cytometry

The beta cell clusters were dissociated using TrypLE™ Express into single cells. Cells were fixed with 4% PFA for 10 min and permeabilizated with cold methanol for 10 min at -20 °C. Primary antibodies were added at a dilution of 1:100 in autoMACs Rinsing Solution (Cat. No. 130-091-222, Miltenyi Biotec) containing 0.5% BSA at 4 °C for 1 hour. Secondary antibodies were added at a dilution of 1:500 at room temperature for 1 hour. Hoechst 33342 (Cat. H3570, Thermo Fisher Scientific) was added 1:2000 dilution together with 2nd antibodies if DNA content was determined. The cells were filtered with BD Falcon 12 x 75 mm tube with a cell strainer cap (Cat. No. 352235, BD) and subsequently analyzed by a flow cytometer. Data was analyzed with the FCS Express 6 Flow software. Negative controls were included by only adding secondary antibodies.

Gene expression analysis using RT-PCR

Insulin-GFP-positive and -negative cells were sorted with flow cytometry. Total RNA was extracted from the sorted cells from different conditions using a RNeasy Mini Kit (Cat. No. 74106, QIAGEN). The total RNA was reverse transcribed into cDNA using iScript™ Reverse Transcription Supermix (Cat. No. 170-8841, Bio-Rad), and the cDNA was then used as template

with SsoFast™ EvaGreen® Supermix (Cat. No. 172-5202, Bio-Rad) for quantitative real-time PCR. The primers used for PCR are listed in Supplemental Table 3.

Western blotting

The sorted GFP-positive and -negative cells with and without APH treatment were lysed with RIPA buffer. Protein was quantified with Pierce BCA protein assay kit. 20 µg of protein was loaded on 4-20% Mini-PROTEAN TGS Precast Protein Gels (Cat. No. 4561094, Bio-Rad) from each condition and ran at 100 V for 1 hour followed by transferring the protein on a PVDF membrane. The membrane was blocked with 3% BSA in TBST for 1h at RT and incubated with primary antibodies diluted in TBST with 3% BSA overnight at -4 °C on a shaker. After washing with TBST, the membrane was incubated with secondary antibodies diluted in TBST with 3% non-fat dry milk powder for 1 h at room temperature. The image was taken with the ChemiDoc Imaging Systems (Bio-Rad).

Proinsulin biosynthesis

The proinsulin biosynthesis was performed on cell clusters at day 27 of differentiation. The iPSC cells were recovered overnight in RPMI 1640 plus GlutaMAX + 1% (v/v) PS + 1% (v/v) B-27 + 10% (v/v) fetal bovine serum + 10 µM zinc sulfate + 10 µg/ml heparin with and without APH. The iPSC clusters were picked in 16mM glucose containing RPMI-1640 medium lacking Cys/Met (Cat. No. R7513, Sigma-Aldrich), washed twice with the same medium and pulse labeled for 15 min with ^{35}S-Cys/Met (300 µCi, Cat. No. NEG072007MC, Perkin Elmer). After pulse, cells were washed once with cold PBS containing 20 mmol/L N-ethylmaleimide (NEM, Cat. No. E3876, Sigma-Aldrich) and then lysed in radioimmunoprecipitation assay buffer (25 mmol/L Tris (Cat.

No. L-27436, Fisher), 1% Triton X-100 (Cat. No. 93443, Sigma), 0.2% deoxycholic acid (Cat. No. BP349, Fisher), 0.1% SDS (Cat. No. 46-040-CI, Corning), 10 mmol/L EDTA (Cat. No. V4231 Promega), and 100 mmol/L NaCl (Cat. No. S9888, Sigma) plus 2mmol/L NEM and a protease inhibitor cocktail (Cat. No. 11836153001, Roche). Cell lysates, normalized to tricholoroacetic acid (Cat. No. BP555-1, Fisher) precipitable counts, were precleared with pansorbin (Cat. No. 507861, Millipore) and immunoprecipitated with anti-proinsulin (Abmart) and protein G-agarose (Cat. No. X1196, Exalpha) overnight at 4°C. Immunoprecipitates were analyzed by nonreducing Tris-tricine-urea-SDS PAGE, with phosphorimaging.

In vitro fluorescence live cell imaging

Live-cell imaging experiments were performed with EVOS FL Auto (Life Technologies). Clusters were plated onto Geltrex in 96-well plate format and cultured for 3 days in EVOS FL Auto chamber. 3 individual clusters were monitored for indicated conditions. Temperature was set to 37 °C, CO_2 was set to 5%. Time-lapse images were taken with 470/22 light cube (Life Technologies) every 30 minutes and compiled to create videos.

Single-cell data analysis

Cell-hashing tags were demultiplexed using HTODemux function (Seurat v3). Empty droplets and doublets were excluded. Cells were excluded by following criteria: 1) total UMI > or < 3 fold of median absolute deviation (MAD); 2) detected genes > or < 3 fold of MAD; 3) detected genes in the first of the bimodal distribution (classified by mclust); 4) mitochondrial gene ratio > 0.15. Cells from two experiment groups (Control and APH) were integrated and clustered to identify cell types

and subpopulations (Seurat v3). The differentially expressed genes between the two groups were defined with 1.28-fold change and 0.05 adjusted p-value.

Transplantation and in vivo assay

The human C-peptide levels in mouse serum were measured between 4-5 pm every two weeks in the fed state. Intraperitoneal glucose tolerance test was performed by fasting overnight and injecting 2 g/kg D-glucose solution at 2 weeks after mouse beta cells were ablated with one high dose (150 mg/kg) of streptozotocin (Cat. No. S0130-1G, Sigma-Aldrich). Blood was collected in heparin-coated tubes at fed state, at fasting state and 30 min after glucose injection. Plasma was collected by centrifuging tubes at 2000 g for 15 min at 4 °C. The supernatants were collected for C-peptide and insulin detection with Mercodia Human Ultrasensitive C-peptide ELISA Kit (Cat. No. 10-1141-01) and Human Insulin ELISA Kit (Cat. No. 10-1113-01). After STZ treatment, mouse C-peptide ELISA kit (Cat. No. 90050, Crystal Chem) was used to determine the elimination of mouse beta cells. Blood glucose levels were measured at fed state, at fasting state (after overnight fasting for 15-16 hours) and every 30 min after glucose injection for 2 hours.

In vivo imaging

Mice were injected with 150 mg/kg D-luciferin (Cat. LUCK-2G, Gold Biotechnology). Subsequently, mice were anesthetized with 3% isoflurane and placed in IVIS Spectrum Optical Imaging System (PerkinElmer) 10 min after injection. Pictures were taken analyzed with Living Image Software. Graft size was determined by bioluminescence intensity.

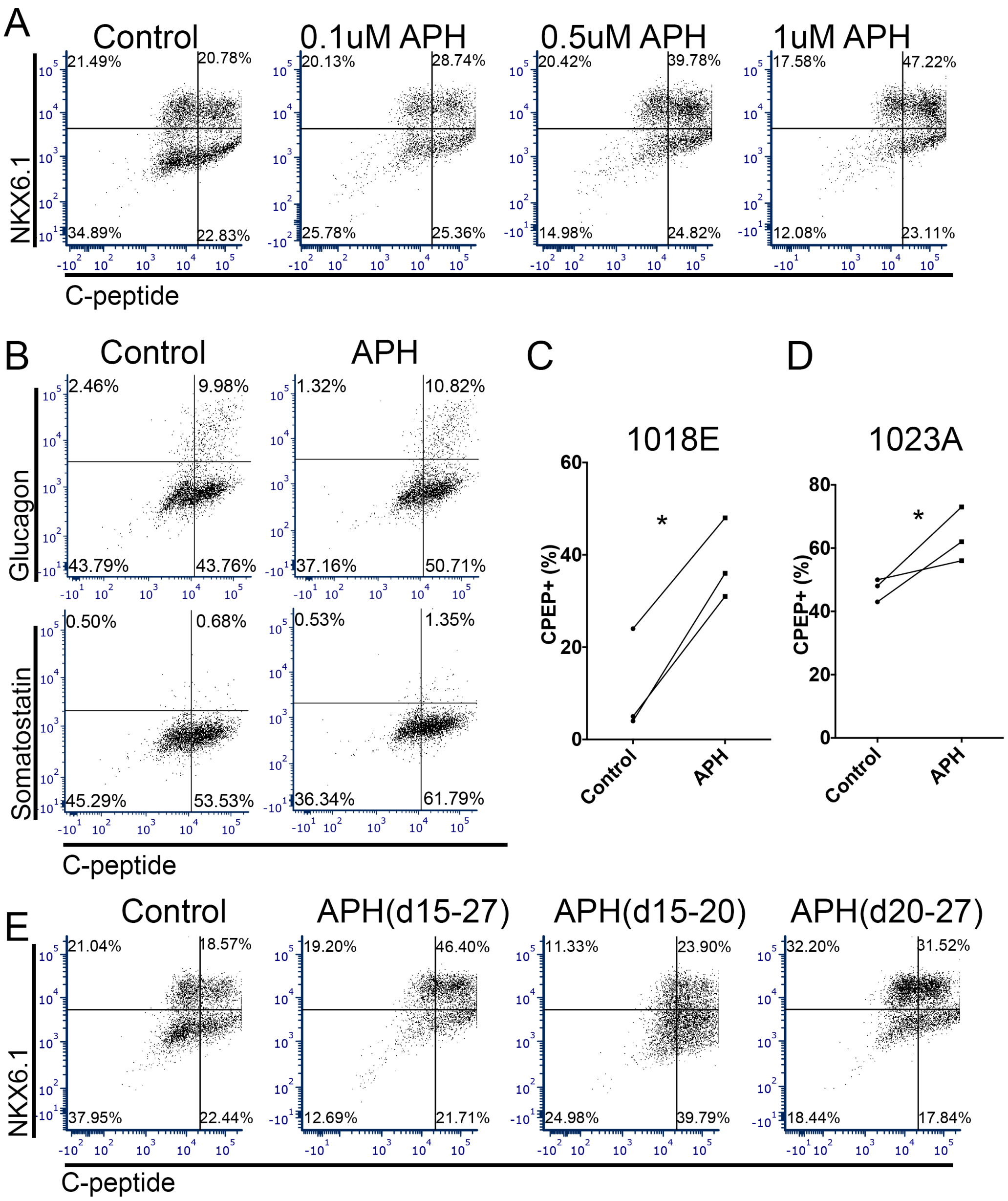

Supplemental Figure 1. Characterization of differentiation efficiency treated with APH with different duration.

(**A**) Quantification of C-peptide and NKX6.1-positive cells derived from MEL-1 cell line on day 27 treated with different concentrations of APH. (**B**) A representative flow cytometry plot for the quantification of C-peptide, glucagon and somatostatin-positive cells in the indicated conditions (**C, D**) Quantification of C-peptide-positive cells at the end of differentiation of two iPSC lines, 1018E and 1023A, depending on the presence or absence of aphidicolin (APH). *: $p<0.05$. (**E**)

Flow cytometry analysis of C-peptide and NKX6.1-positive cells derived from MEL-1 cell line on day 27 with different duration of APH treatment. (**E**) Flow quantification of TUNEL and C-peptide-positive cells of MEL-1 cell line in indicated conditions on day 17 (2 days after treatment). Related to Figure 2.

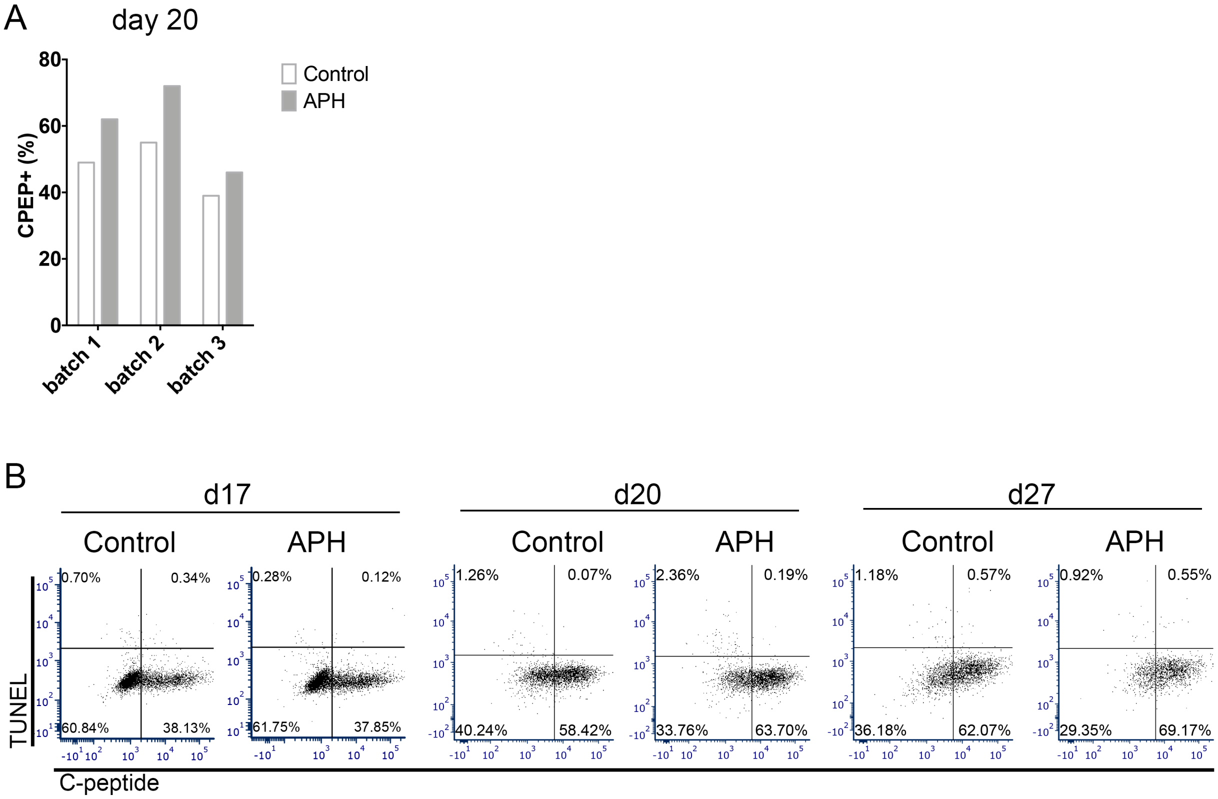

Supplemental Figure 2. Characterization of cells treated with APH at different time points. **(A)** Percentage of C-peptide-positive cells from 3 batches of differentiation using MEL-1 cell line with and without APH treatment. (**B**) A representative flow cytometry analysis to determine the percentage of C-peptide and TUNEL-positive cells on d17, d20 and d27 with and without APH treatment.
Related to Figure 2.

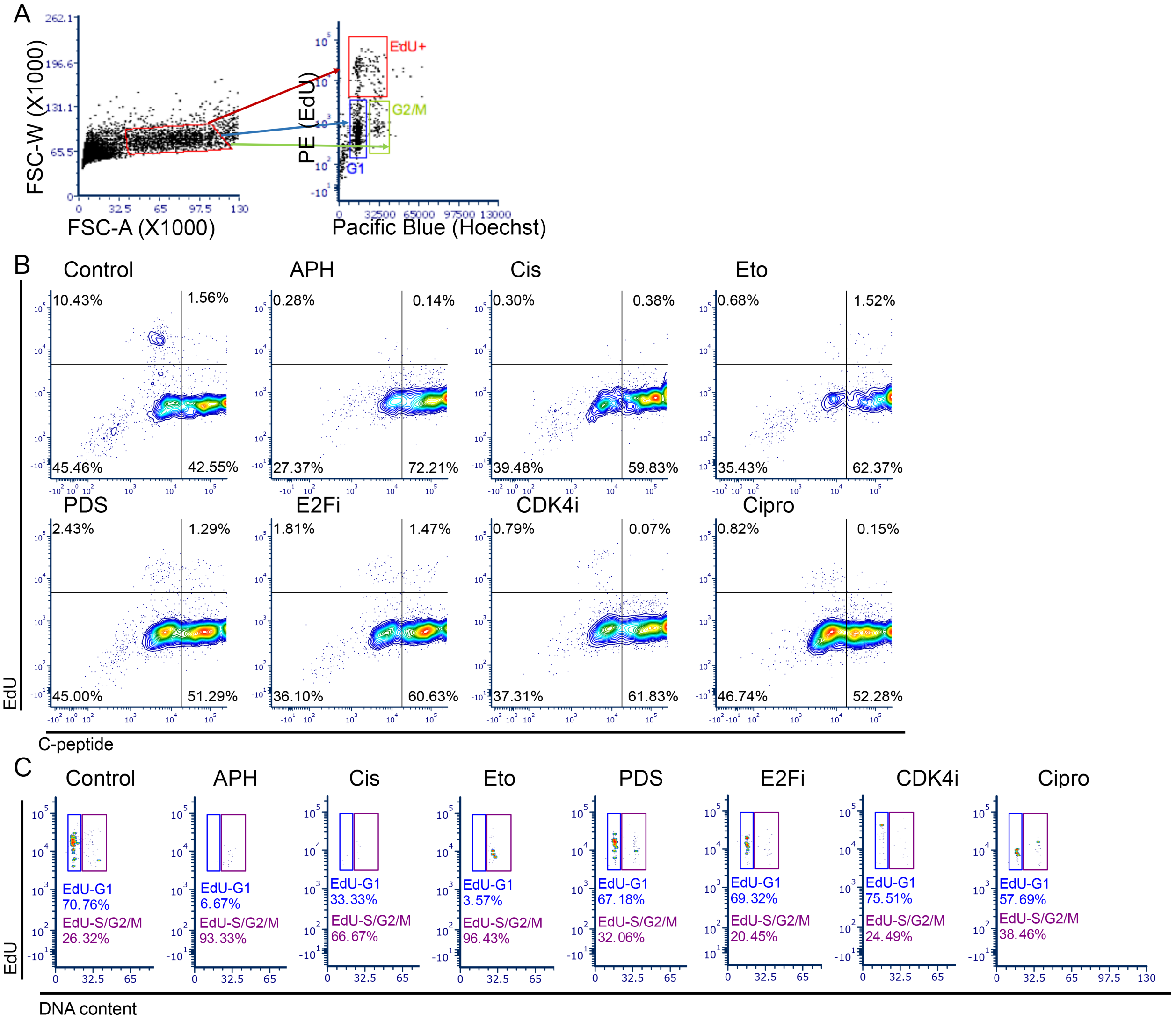

Supplemental Figure 3. Analysis of cell cycle progression of day 27 differentiated cells treated with indicated compounds.

All experiments were performed with the MEL-1 cell line on day27 of differentiation.

(A) Gating strategy for cell cycle analysis. Single cells were gated with FSC-A (Forward scatter area) and FSC-W (Forward scatter width). Cells in S were gated based on EdU positivity, cells in G1 and G2/M were gated as EdU-negative cells based on different DNA content indicated by DNA staining with Hoechst. (**B**) Flow plot of C-peptide and EdU labeled cells in indicated conditions. (**C**) Cells treated with indicated compounds were labeled with EdU for 2 hours on day 26, and analyzed 1 day later for cell cycle distribution. The proportion of EdU-positive cells in G1 phase (EdU-G1) and S/G2/M (EdU-S/G2/M) phase was quantified. APH: Aphidicolin (1 μM); Cis: Cisplatin (2.5 μM); Eto: Etoposide (2 μM); PDS: Pyridostatin (10 μM); E2Fi: E2F inhibitor (10 μM); CDK4i: CDK4 inhibitor (2 μM); Cipro: Ciprofloxacin (100 μM).

Related to Figure 3.

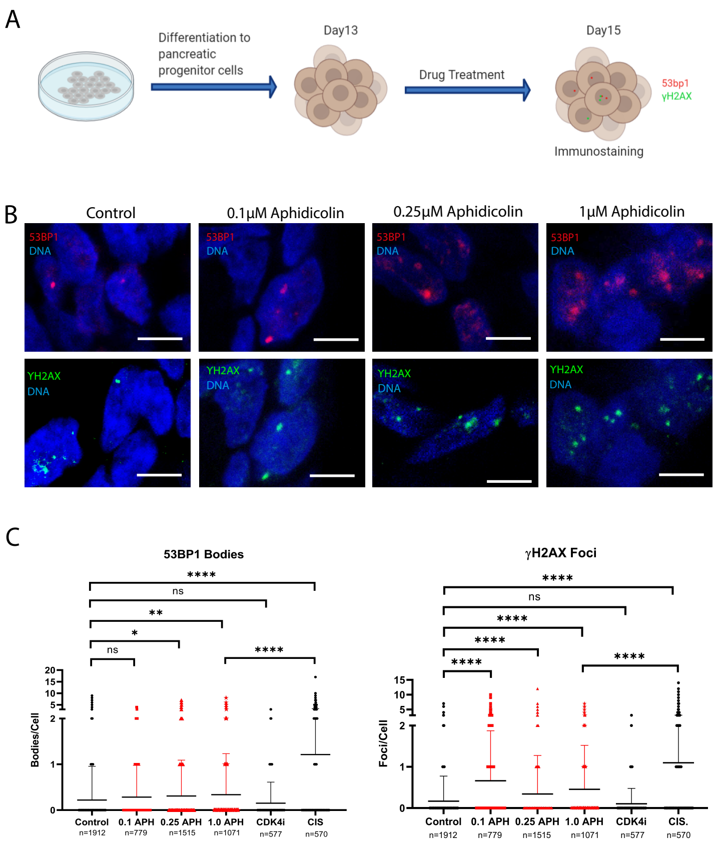

Supplemental Figure 4. Aphidicolin treatment modestly increases 53BP1 bodies in differentiating pancreatic progenitor cells.

(**A**) Experimental schematic. ES cell line MEL-1 was differentiated to pancreatic progenitor cells and exposed to indicated concentrations of aphidicolin or 2.5 μM cisplatin or 2 μM CDK4i for 48hr from day 13 to day 15.

(**B**) Immunostaining for 53BP1 and γH2AX performed on cells with or without indicated treatment on day 15 of differentiation.

(**C**) Quantification of 53BP1 bodies and γH2AX foci.

Scale bar: 5μm. Statistical analysis was performed using one-way ANOVA (*p<0.05, **p<0.005, ****p<0.001).

Related to Figure 3.

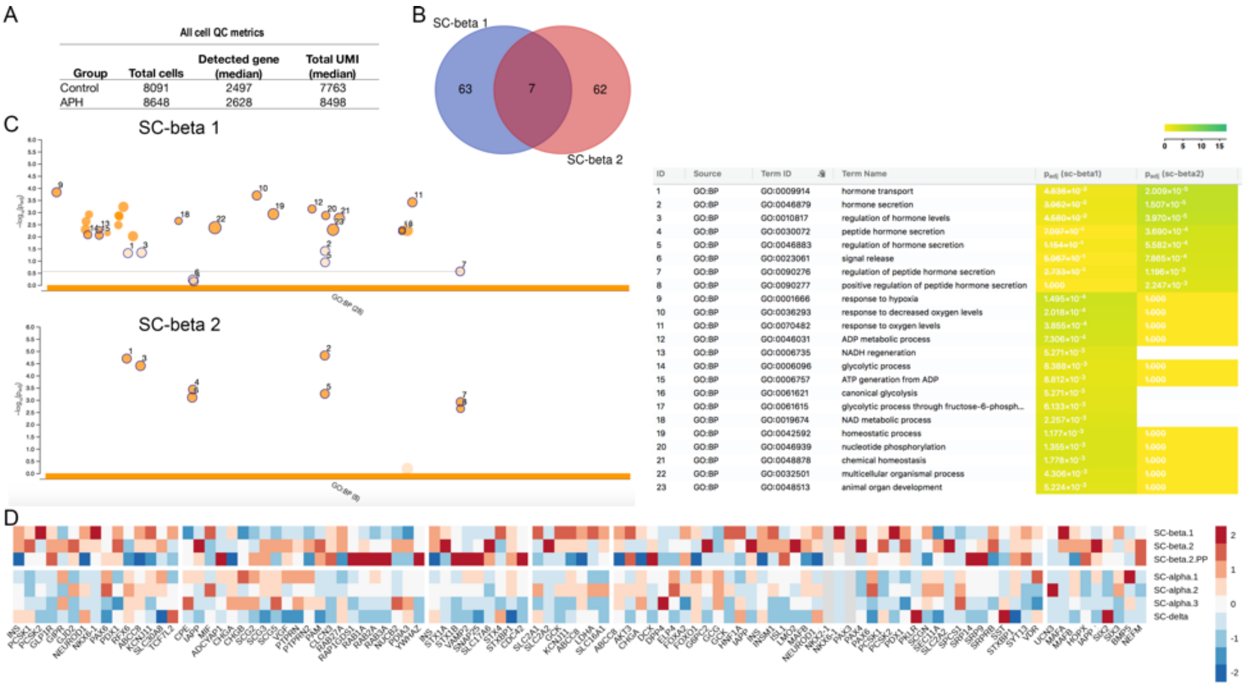

Supplemental Figure 5. Characteristics of SC-beta 1 cells and SC-beta 2 cells derived from MEL-1 cell line with single cell RNA sequencing.

(A) Cell quality control metrics of single cell sequencing. (**B**) Venn Diagram illustrates the numbers of gene expressed in SC-beta 1 and SC-beta 2 clusters. (**C**) Gene ontology enrichment analysis of genes expressed in SC-beta 1 and SC-beta 2 and identification of significantly enriched GO terms. Genes expressed in SC-beta 1 are enriched in the biological process of glycolytic process and response to hypoxia, whereas genes in SC-beta 2 are highly enriched in the process of hormone transport and secretion. (**D**) Heatmap of relative expression of genes in beta cell identity, maturation, glucose metabolism and insulin processing and releasing between identified endocrine population. Expression level was centered and scaled by each gene. Related to Figure 5.

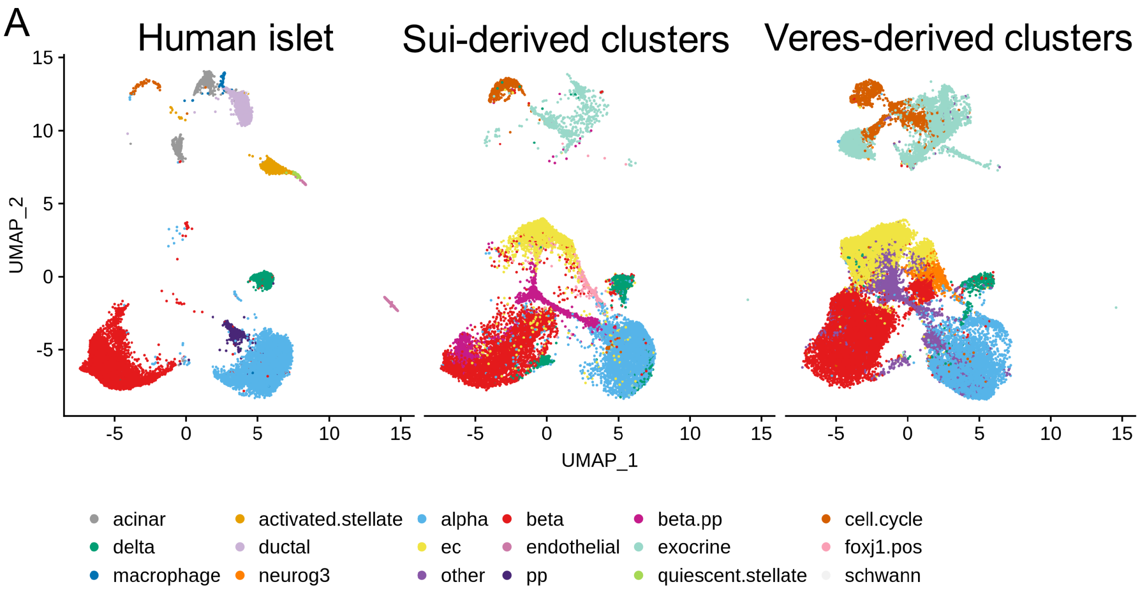

Supplemental Figure 6. Single cell RNA sequencing analysis of human islet, Sui-derived clusters and Veres-derived clusters

(**A**) Cell population mapping from human islets, MEL-1 stem cell-derived cells in this study (Sui) and of another study (Veres) (Veres et al., 2019). We use integrative analysis (Seurat v3) to identify cell types / states from different batches by finding “anchors” (i.e., common molecular features) across data sets.

Related to Figure 4.

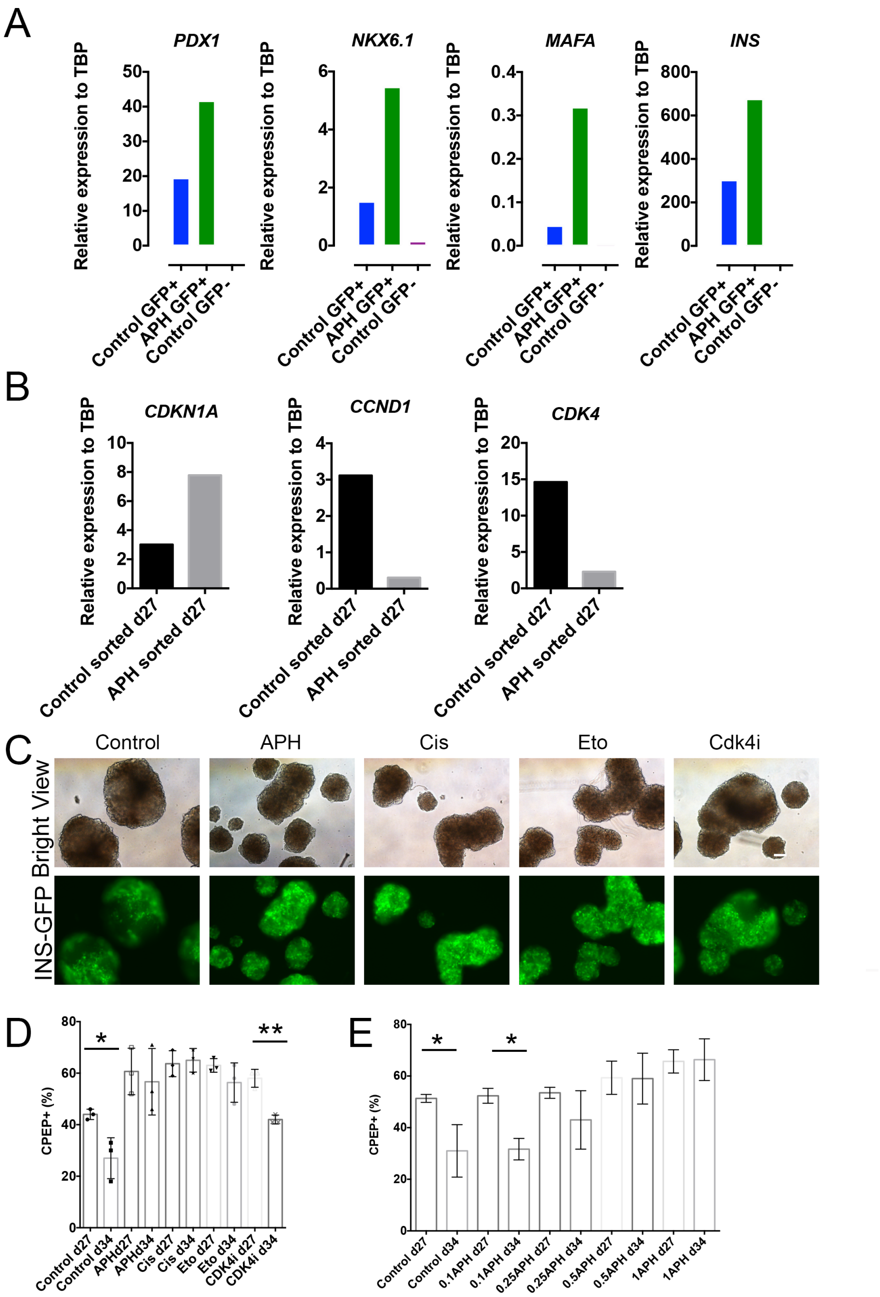

Supplemental Figure 7. Stabilization of insulin-producing cell identity with Aphidicolin (APH), Cisplatin (Cis) and Etoposide (Eto). All data is derived from the MEL-1 cell line. (**A**) Beta cell gene expressions of sorted cells by RT-PCR. (**B**) Expression of cell cycle inhibitor (*CDKN1A*) and cell cycle activators (*CCND1* and *CDK4*) in the GFP-positive cells sorted from APH and control group analyzed by RT-PCR. (**C**) Expression of insulin-GFP in derived clusters on day 34, 7 days after they were released from the indicated compounds on day 27. Scale bar: 100 μm. (**D**) Percentage of C-peptide-positive cells on day 27 and day 34 after releasing from indicated compounds from day 27. *: $p<0.05$; **: $p<0.01$. (**E**) Percentage of C-peptide-positive cells at day 27 and day 34 after releasing from different concentrations of Aphidicolin from day 27. *: $p<0.05$.

Related to Figure 5.

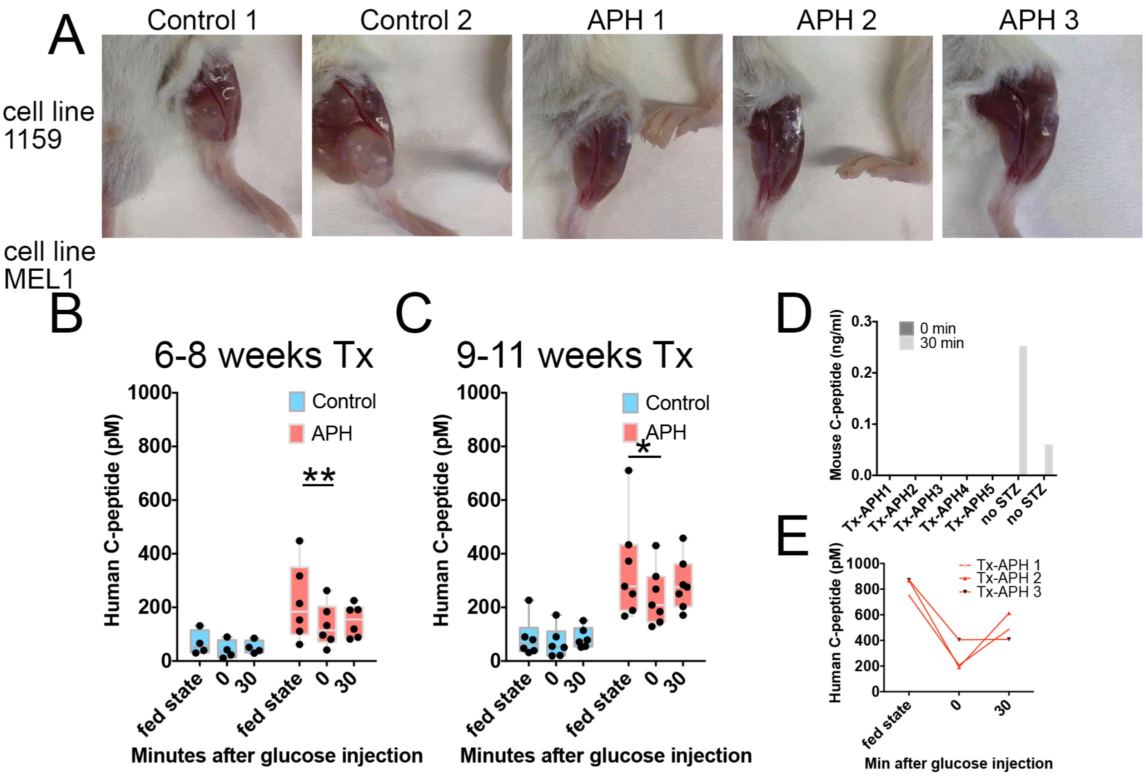

Supplemental Figure 8. APH pretreatment prevents graft growth after transplantation; grafted cells show glucose-dependent human C-peptide secretion.

(**A**) Picture of graft site 1159 iPSC-clusters transplanted for 6 months. (**B**) Human C-peptide serum concentration in mice at 6-8 weeks after transplantation of MEL-1 cell line derived clusters and (**C**) 9-11 weeks after transplantation with cells treated with APH (APH) and control cells (MEL-1) at fed state, fasting and 30 min after glucose injection. *: $p<0.05$; **: $p<0.01$. (**D**) Serum mouse C-peptide concentrations of STZ-treated mice transplanted with APH-treated cells at fasted state and 30 min after glucose injection. (**E**) Serum human C-peptide concentrations of STZ-treated mice transplanted with APH-treated cells at fed state, at fasted state and 30 min after glucose injection.

Related to Figure 6.

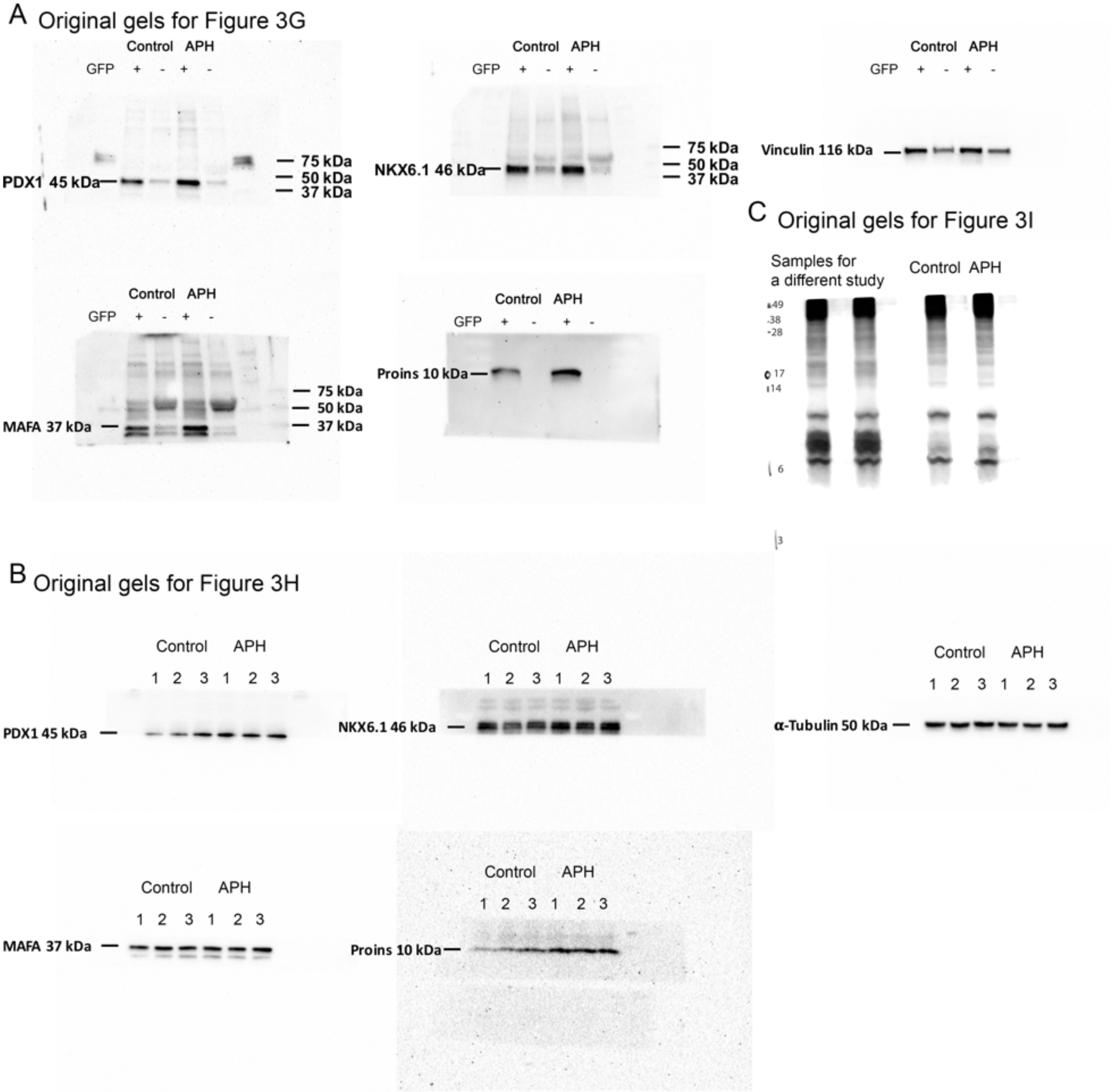

Supplemental Figure 9. Original uncut blots

(**A, B**) Uncut western blot membranes with size ladder and protein size indicated. (**C**) A blot of immunoprecipitated 35S-Cys/Met labeled newly synthesized proinsulin with size ladder. The levels were normalized to TCA precipitable counts.

Related to Figure 4.

A

Amelogenin	X	Y	D18S51	14	16
vWA	14	19	Penta E	7	
D8S1179	13		D5S818	11	12
TPOX	8	11	D13S317	11	14
FGA	19	23	D7S820	9	10
D3S1358	16	17	D16S539	11	12
THO1	9.3		CSF1PO	10	11
D21S11	29	31.2	Penta D	9	10

B

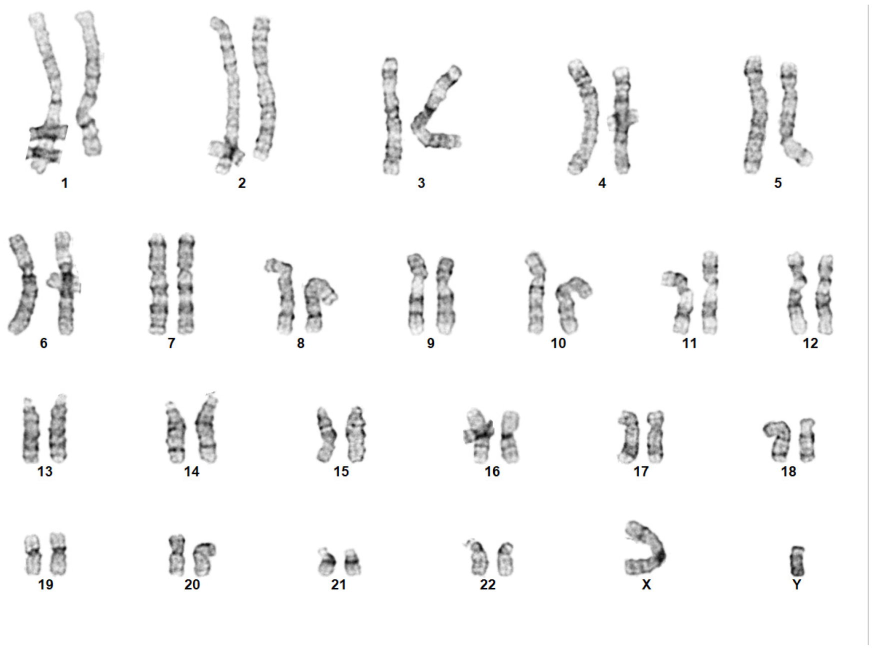

Supplemental Figure 10. 1159B cell line characterization.
(A) The DNA STR profile of cell line 1159B. (B) Normal human male karyotype.

Supplemental Table 1. Information of human pluripotent stem cell lines

Stem cell line ID	Reprograming method	Origin	Sex	Diagnosis	Reference
MEL-1 ($INS^{GFP/w}$ $GAPDH^{Luc/w}$ hESC)	n/a	Inner cell mass	Male	n/a	(Micallef et al., 2012)
1023A	mRNA	fibroblast	Male	Healthy control	(Paull et al., 2013)
1018E	mRNA	fibroblast	Female	T1D	(Johannesson et al., 2014)
1159 B	mRNA	fibroblast	Female	Healthy control	(Sui et al., 2018)

Supplemental Table 2. Information of compounds used to interfere with cell cycle progression.

Compounds	Mechanism of Action	Concentration
Palbociclib Isethionate (CDK4i) (Cat. No. S1579, Selleck Chemicals)	arrests cells in early G1 phase	2 µM
Ciprofloxacin (Cat. No. S2027 Selleck Chemicals)	prevents entry into S phase	100 µM
E2F inhibitor (Cat. No. 324461 EMD Millipore)	prevents entry into S phase	10 µM
Cisplatin (Cat. No. 232120, EMD Millipore)	interferes with S-phase progression and arrests cells at G0/G1 phase	2.5 µM
Etoposide (Cat. No. E55500, Research Products International)	prevents the unwinding of the DNA helix during replication and transcription and arrests cells in the S and G2 phases	2 µM
Pyridostatin (Cat. No. S7444, Selleck Chemicals)	promotes the formation of G4 structures to induce replication-fork stalling, thereby affecting S-phase progression	10 µM
Aphidicolin (Cat. No. A0781, Sigma-Aldrich)	inhibits G1 to S phase transition and S-phase progression in a concentration dependent manner	0.1 µM, 0.25 µM, 0.5 µM and 1 µM

Supplemental Table 3. Primers used for quantitative real-time PCR at different stage during the differentiation.

Gene	Forward	Reverse
PDX1	CCCTGGGTGACCACTAAACC	CACAGCCTCTACCTCGGAAC
NKX6.1	ATTCGTTGGGGATGACAGAG	CGAGTCCTGCTTCTTCTTGG
MAFA	CTTCAGCAAGGAGGAGGTCA	GCTCTGGAGTTGGCACTTCT
INS	TTCTACACACCCAAGACCCG	CAATGCCACGCTTCTGC
CDKN1A	ACAGCAGAGGAAGACCATGTG	AGTGGTAGAAATCTGTCATGCTG
CCND1	CAATGACCCCGCACGATTTC	ATGAACTTCACATCTGTGGCA
CDK4	ACACCCGTGGTTGTTACACT	ACAGAAGAGAGGCTTTCGACG
TBP	TGTGCACAGGAGCCAAGAGT	ATTTTCTTGCTGCCAGTCTGG

Supplemental Table 4. Primary antibody list

Antibody	Species	Dilution	Company	Catalog number
PDX1	Goat	1:100	R&D Systems	AF2419
NKX6.1	Mouse	1:300	Developmental Studies Hybridoma Bank	F55A10
C-peptide	Rat	1:100	Developmental Studies Hybridoma Bank	GN-ID4
Glucagon	Guinea Pig	1:200	Takara	M182
MAFA	Rabbit	1:100	Abcam	ab26405
Somatostatin	Rabbit	1:1000	Millipore	AB5494
53BP1	Rabbit	1:200	Santa Cruz Biotechnology	SC-22760
Ki67	Rabbit	1:200	Abcam	AB16667

Supplemental Table 5. Secondary antibody list

Antibody	Dilution	Company	Catalog number
Donkey Anti-Rat Alexa Fluor® 488	1:500	Jackson ImmunoResearch Laboratories	712-545-153
Donkey Anti-Guinea Pig Alexa Fluor® 647	1:500	Jackson ImmunoResearch Laboratories	706-605-148
Donkey Anti-Rabbit Alexa Fluor® 555	1:500	Life Technologies	A-31572
Donkey Anti-Mouse Alexa Fluor® 488	1:500	Life Technologies	A-21202
Donkey Anti-Mouse Alexa Fluor® 555	1:500	Life Technologies	A-31570
Donkey Anti-Rabbit Alexa Fluor® 488	1:500	Life Technologies	A-21206
Goat Anti-Rat Alexa Fluor® 555	1:500	Life Technologies	A-21434

Supplemental Table 6. Quantification of positive cells for indicated markers in grafts.

Graft	Total cells	Ki67+	Ki67+/Total (%)	Graft	INS+	INS+MAFA+	INS+MAFA+/INS+ (%)
Control-1	421	9	2.1	Control-1	33	18	54.5
Control-2	639	112	17.5	Control-2	290	38	13.1
	630	16	2.5		301	43	14.3
	755	63	8.3	APH-1	496	109	22
	487	78	16		319	50	15.7
APH-1	1187	22	1.9	APH-2	328	91	27.7
	1213	19	1.6				
APH-2	745	20	2.7				
	1140	36	3.2				
APH-3	711	5	0.7				
	492	6	1.2				

Supplemental Table 7. Gene list of differentially expressed genes between SC-beta 1 and SC-beta 2 populations

Gene	SC-beta.1.umi.ave	SC-beta.2.umi.ave
PCSK1N	28.83	50.94
ERO1LB	3.57	8.36
PCP4	1.21	4.40
HADH	0.27	1.72
RTN1	0.62	2.41
SCG5	10.46	18.47
IAPP	2.85	42.06
MIF	47.09	31.75
SLC2A1	5.49	1.09
APLP1	1.94	4.03
STMN2	5.69	11.58
LPPR5	0.30	1.01
STMN1	5.14	8.84
SYNPO	1.47	0.18
DSP	6.00	2.69
GNG3	1.05	3.59
ASB9	0.08	0.79
IRX2	1.72	0.45
LDHA	9.33	3.90
IGFBP2	2.95	0.72
SLC14A1	1.54	0.14
CPE	6.70	11.39
NGFRAP1	6.47	9.91
UCHL1	2.47	4.21
CCND1	1.91	0.25
PIPOX	0.36	1.13
TTR	132.43	215.89
MLLT11	2.92	4.93
SPOCK3	0.28	1.03
APOC1	6.36	2.71
TMED3	1.97	3.56
CDH8	0.38	1.08
PLD3	8.21	13.04
TMEM59L	1.08	2.37
ACVR1C	3.60	6.17
TPI1	19.38	11.52
ATP5E	7.45	11.51

C5orf38	2.04	0.74
PCDH7	0.46	1.23
NEFM	2.45	8.57
MARCKSL1	4.71	7.07
NEAT1	4.50	0.82
TUSC3	2.79	4.30
VIM	3.67	0.57
BEX1	7.17	10.26
CADM1	0.60	1.30
ZNF395	2.50	0.70
HSF4	1.20	0.31
HIST1H2AC	0.65	1.51
IRX1	0.97	0.19
TUBB2B	1.95	3.69
APOE	5.91	2.69
TUBA1A	9.19	14.03
ENSG00000198786	21.98	14.02
DHRS2	0.57	1.59
LHFP	0.09	0.47
UBE2E3	0.62	1.24
IGFBP3	6.98	1.75
ENO1	9.21	5.08
ALDOA	9.07	5.14
TMEM141	3.83	1.53
DCX	1.01	1.98
INHA	2.20	0.57
SLC16A3	1.26	0.19
ADM	1.52	0.18
IGSF1	0.19	0.63
SCG3	3.75	5.54
LRRTM4	0.07	0.42
CITED2	7.09	2.04
NSG1	0.16	0.60
PLK2	0.27	0.79
HPCA	0.79	1.52
LOC728392	1.91	3.21
TMEM163	1.64	0.43
ASIC4	0.32	0.79
TUBB2A	0.56	1.24
ITM2C	0.87	1.56

LY6H	1.17	2.61
LMO2	0.59	1.38
HLA-B	1.66	0.29
SAMD11	0.23	0.69
MIAT	4.65	1.74
BTBD17	0.88	1.94
FTL	66.56	95.17
NDN	1.41	2.49
HILPDA	5.54	0.51
MAGED2	2.40	3.61
RTN4	7.12	4.04
SLC30A8	2.55	4.15
AK4	1.07	0.27
CRIP2	1.28	2.20
LINC00643	0.46	1.02
TAGLN2	6.39	3.81
CHODL	0.34	1.01
LGALS3	0.43	0.02
PPIB	5.37	8.20
ISL1	3.30	5.03
MYEOV2	3.47	4.88
NPDC1	2.28	3.36
PDLIM4	1.92	0.61
NEFL	0.57	2.22
ATP6AP2	1.29	2.18
BHLHE40	0.49	0.06
SERPINI1	0.36	0.84
STC2	1.74	0.47
ST6GALNAC5	0.81	1.38
NME1	1.27	2.02
TUBB3	3.28	5.04
CST3	6.33	8.87
GAP43	0.48	1.29
PLAGL1	0.59	1.14
CALR	2.97	4.44
PCSK1	0.68	1.36
TTC3	2.44	3.53
MAP1B	4.83	6.52
SLC9A3R1	0.93	1.57
GSTP1	4.23	2.27

C20orf27	0.71	1.27
VTN	1.44	2.48
PEPD	0.41	0.85
WFDC2	1.54	0.52
INA	0.84	1.38
SDF2L1	0.78	1.39
HOPX	0.88	1.65
ERP29	3.47	4.87
ECEL1	0.78	1.44
TAGLN3	0.51	0.97
TXNIP	1.33	0.48
CDHR3	0.41	0.05
IFI6	14.61	19.91
PAM	0.89	1.69
TMEM190	3.43	1.48
FAM46A	0.96	1.68
SFRP1	0.49	0.10
MANF	1.40	2.25
OBSL1	1.97	0.89
STC1	1.09	0.30
B2M	7.42	3.79
HES1	1.36	0.26
RBP4	0.13	0.76
SLC22A17	1.31	2.12
CDO1	0.88	0.18
CXCR4	1.30	0.36
SCG2	1.51	2.60
PHLDA2	0.25	0.72
PTPRN	2.35	3.42
C4orf3	3.64	2.01
PFKP	1.11	0.43
EPAS1	1.18	0.54
SYT7	2.20	1.21
KCTD12	0.82	0.25
GCK	2.39	1.23
PGK1	3.51	2.10
PCSK2	2.22	1.12
C10orf10	1.09	0.25
SEC11C	1.85	2.69
CDCA7L	0.71	0.14

FGF18	0.51	0.09
H1F0	2.00	0.96
MT2A	7.92	1.23
HLA-C	0.63	0.16
ASPH	1.28	2.14
GPI	2.25	1.28
SLC2A3	0.89	0.24
VSTM2L	0.45	0.10
HLA-E	0.56	0.15
PPP1R1A	7.96	5.14
SPINK1	2.05	0.73
FGF11	0.49	0.14
ST3GAL6	0.76	0.28
WDR45B	2.34	1.33
BNIP3L	2.07	1.02
G0S2	1.58	0.58
DMKN	1.42	0.63
S100A11	2.28	1.23
P4HA1	0.82	0.29
F3	0.87	0.24
HSPB1	1.14	0.45
FEV	6.49	3.61
MSRA	0.61	0.17
IRF2BP2	1.92	1.06
HMGB3	2.56	0.71
APOC3	1.79	0.55
PDCD4	1.89	0.91
PPP1R3C	0.66	0.16
IL17RB	0.41	0.07
TMEM56	0.63	0.23
NDRG1	0.55	0.14
CCND2	0.85	0.37
PLOD2	0.84	0.30
MTFP1	0.82	0.30
CDC42EP3	1.49	0.74
FBXO17	2.84	1.63
CCDC151	0.62	0.23
MT1E	4.18	0.74
AF070581	2.26	1.32
MT1G	9.68	1.41

NCKAP5	0.89	0.36
KISS1R	0.86	0.35
GCLC	0.57	0.21
TM4SF4	0.92	0.27
MT1X	15.19	2.55
PLCXD1	0.58	0.18
VGF	0.50	1.18
H2AFJ	1.46	0.79
INSIG2	0.70	0.26
ZBTB38	0.78	0.34
MT1F	3.38	1.25
IER2	0.82	0.36
CGA	0.31	2.35
TMEM37	1.94	1.23
S100A10	0.95	0.49
GADD45G	0.99	0.47
LAPTM4B	1.34	0.75
JUNB	1.71	1.03

Supplemental Table 8. Gene list of differentially expressed genes between Control and APH treated cells in SC-beta 1 and SC-beta 2 populations

Cluster	Comparison	Gene	logFC	Cell ratio in control	Cell ratio in APH	Expr in control	Expr in APH
SC-beta.1	Control vs. APH	*GCG*	0.43	0.997	0.993	21.78	13.86
SC-beta.1	Control vs. APH	*HILPDA*	0.40	0.545	0.477	6.83	4.24
SC-beta.1	Control vs. APH	*CITED2*	0.29	0.778	0.712	8.24	5.94
SC-beta.1	Control vs. APH	*DDC*	0.28	0.69	0.59	3.06	2.06
SC-beta.1	Control vs. APH	*VIM*	0.25	0.612	0.529	4.24	3.10
SC-beta.1	Control vs. APH	*RTN4*	0.21	0.941	0.929	7.99	6.26
SC-beta.1	Control vs. APH	*TMEM190*	0.21	0.718	0.657	3.89	2.97
SC-beta.1	Control vs. APH	*INHA*	0.20	0.596	0.511	2.52	1.88
SC-beta.1	Control vs. APH	*B2M*	0.20	0.891	0.847	8.26	6.58
SC-beta.1	Control vs. APH	*MLLT11*	0.18	0.791	0.734	3.28	2.56
SC-beta.1	Control vs. APH	*SKP1*	0.18	0.877	0.853	4.26	3.41
SC-beta.1	Control vs. APH	*TAGLN2*	0.17	0.944	0.905	7.03	5.76
SC-beta.1	Control vs. APH	*TPI1*	0.17	0.993	0.981	21.09	17.66
SC-beta.1	Control vs. APH	*GSTP1*	0.17	0.863	0.805	4.66	3.79
SC-beta.1	Control vs. APH	*SLC14A1*	0.16	0.466	0.363	1.75	1.34
SC-beta.1	Control vs. APH	*HSPA8*	0.15	0.895	0.861	5.87	4.89
SC-beta.1	Control vs. APH	*TTR*	-0.16	1	1	122.11	142.78
SC-beta.1	Control vs. APH	*MT-RNR2*	-0.16	0.969	0.981	20.26	23.92
SC-beta.1	Control vs. APH	*MT-CO2*	-0.16	0.997	0.997	62.91	74.21
SC-beta.1	Control vs. APH	*MT-CO1*	-0.17	0.994	0.997	68.86	81.98
SC-beta.1	Control vs. APH	*ONECUT2*	-0.19	0.417	0.513	0.95	1.35
SC-beta.1	Control vs. APH	*MT-CO3*	-0.19	0.986	0.994	35.51	43.04
SC-beta.1	Control vs. APH	*MT-TP*	-0.19	0.483	0.599	1.65	2.20
SC-beta.1	Control vs. APH	*MT-ND1*	-0.21	0.981	0.991	19.27	24.08
SC-beta.1	Control vs. APH	*VTN*	-0.21	0.377	0.475	1.19	1.70
SC-beta.1	Control vs. APH	*MT-ND3*	-0.21	0.979	0.989	24.43	30.47
SC-beta.1	Control vs. APH	*MT-TE*	-0.23	0.465	0.588	1.80	2.53
SC-beta.1	Control vs. APH	*ERO1LB*	-0.25	0.684	0.762	3.01	4.13
SC-beta.1	Control vs. APH	*GNAS*	-0.32	0.968	0.976	20.74	28.85
SC-beta.2	Control vs. APH	*GCG*	0.55	0.995	0.995	18.92	10.55
SC-beta.2	Control vs. APH	*APOC1*	0.37	0.8	0.714	3.58	2.17
SC-beta.2	Control vs. APH	*TMEM190*	0.29	0.606	0.531	1.93	1.19
SC-beta.2	Control vs. APH	*APOE*	0.21	0.843	0.799	3.18	2.38
SC-beta.2	Control vs. APH	*B2M*	0.21	0.912	0.879	4.42	3.40
SC-beta.2	Control vs. APH	*FEV*	0.21	0.835	0.779	4.21	3.24
SC-beta.2	Control vs. APH	*CCND1*	0.21	0.167	0.082	0.41	0.15
SC-beta.2	Control vs. APH	*INHA*	0.19	0.326	0.239	0.76	0.46
SC-beta.2	Control vs. APH	*C5orf38*	0.17	0.518	0.39	0.93	0.63
SC-beta.2	Control vs. APH	*IGFBP2*	0.15	0.444	0.355	0.88	0.62
SC-beta.2	Control vs. APH	*SMYD3*	-0.16	0.436	0.548	0.59	0.87
SC-beta.2	Control vs. APH	*IAPP*	-0.18	0.785	0.859	37.52	44.92
SC-beta.2	Control vs. APH	*PCP4*	-0.18	0.776	0.851	3.83	4.76
SC-beta.2	Control vs. APH	*NPW*	-0.27	0.357	0.485	0.81	1.37

References:

Johannesson, B., Sagi, I., Gore, A., Paull, D., Yamada, M., Golan-Lev, T., Li, Z., LeDuc, C., Shen, Y., Stern, S., *et al.* (2014). Comparable frequencies of coding mutations and loss of imprinting in human pluripotent cells derived by nuclear transfer and defined factors.

Micallef, S.J., Li, X., Schiesser, J.V., Hirst, C.E., Yu, Q.C., Lim, S.M., Nostro, M.C., Elliott, D.A., Sarangi, F., Harrison, L.C., *et al.* (2012). INS(GFP/w) human embryonic stem cells facilitate isolation of in vitro derived insulin-producing cells. Diabetologia *55*, 694-706.

Paull, D., Emmanuele, V., Weiss, K.A., Treff, N., Stewart, L., Hua, H., Zimmer, M., Kahler, D.J., Goland, R.S., Noggle, S.A., *et al.* (2013). Nuclear genome transfer in human oocytes eliminates mitochondrial DNA variants. Nature *493*, 632-637.

Sui, L., Danzl, N., Campbell, S.R., Viola, R., Williams, D., Xing, Y., Wang, Y., Phillips, N., Poffenberger, G., Johannesson, B., *et al.* (2018). beta-Cell Replacement in Mice Using Human Type 1 Diabetes Nuclear Transfer Embryonic Stem Cells. Diabetes *67*, 26-35.

Veres, A., Faust, A.L., Bushnell, H.L., Engquist, E.N., Kenty, J.H., Harb, G., Poh, Y.C., Sintov, E., Gurtler, M., Pagliuca, F.W., *et al.* (2019). Charting cellular identity during human in vitro beta-cell differentiation. Nature *569*, 368-373.
